# Application of Systems Genetics to Investigate Molecular Pathology of Early‐Onset Colorectal Cancer

**DOI:** 10.1111/cbdd.70379

**Published:** 2026-08-11

**Authors:** Minjae J. Kim, Sydney L. Carr, Vishnutheertha Kulkarni, Mary‐Louise Manchadi

**Affiliations:** ^1^ University of Tennessee Health Science Center College of Medicine Memphis Tennessee USA; ^2^ University of Queensland Medical School Brisbane Queensland Australia

**Keywords:** biomarker discovery, discovering drug targets, drug research, early‐onset colorectal cancer, genomics, mechanisms of disease, multi‐omics, proteomics, transcriptomics

## Abstract

Colorectal cancer (CRC) is the second most common cause of cancer‐related deaths in the United States. The incidence of early‐onset CRC (EOCRC) has been rising in the past few decades. Given the multifaceted nature of EOCRC, the genetic and environmental factors contributing to this malignancy have remained elusive. Systems genetics is a multi‐omics tool that analyzes multiple genes as a collective network. Transcriptional correlation, protein–protein interaction network, functional annotation, and drug‐gene interaction analyzes enable investigators to explore the functional roles of target genes and aid with discovery of novel diagnostic biomarkers and potential therapeutics. To clarify the objective of this work, this manuscript serves three purposes: a narrative review of recent systems genetics applications in EOCRC research, a step‐by‐step protocol for conducting these analyses, and a discussion of current advances in systems genetics to address limitations in generalizability and context dependence. Through this manuscript, we envision a future in which emerging oncology researchers adopt these tools into their investigations of EOCRC and other malignancies.

## Graphical abstract

Early‐onset colorectal cancer (EOCRC) presents a complex molecular landscape that requires integrated multi‐omics approaches. This article reviews current applications of systems genetics in EOCRC research and provides a step‐by‐step protocol for network‐based analyses featuring *PIK3CA* to demonstrate practical application for researchers of all levels. Finally, we discuss how the integration of artificial intelligence models is overcoming current limitations to enhance the speed and generalizability of systems genetics research in EOCRC.

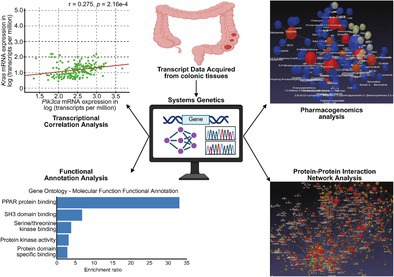

**Abbreviations**
AI: artificial intelligence
CRC: colorectal cancer
DEG: differentially expressed gene
EOCRC: early‐onset colorectal cancer
GBM: gradient boosting machine
GWAS: genome‐wide association study
KEGG: Kyoto Encyclopedia of Genes and Genomes
LASSO: least absolute shrinkage and selection operator
LLM: Large Language Model
LOCRC: late‐onset colorectal cancer
MSI: microsatellite instability
NGS: next‐generation sequencing
PPI: protein–protein interaction

## 1 Introduction

Colorectal cancer (CRC) is the second most common cause of cancer‐related deaths and ranks first among adults younger than 50 years of age, with a projected incidence of 158,850 cases in 2026 (Siegel et al. 2026). Given its rising incidence, current prophylactic measures for this malignancy include colonoscopy every 10 years, a fecal DNA test every 3 years, and a fecal immunochemical/blood test every 1 year (Meester et al. 2026). Notably, the age of screening has been trending downwards, targeting younger demographics. In fact, the United States Preventive Services Task Force (USPSTF) changed the guideline for CRC screening from age 50 to 45 in 2021 (Meester et al. 2026). This downward trend in screening initiation reflects current demographic changes in CRC. Notably, early‐onset CRC (EOCRC), affecting patients below the age of 50, is rising, with incidence increasing by 3% annually among adults aged 20–49 years, predominantly in the distal colon and rectum (Siegel et al. 2026). Given the multifaceted nature of this malignancy, several factors have been explored to explain this downward trend. One possibility is increased production and consumption of ultra‐processed foods, which have been associated with an increased risk of EOCRC in women (Wang et al. 2026). Another possibility is changes in gut microbiome, and EOCRC was found to harbor unique microbial and host‐environmental features compared to late‐onset CRC (LOCRC) (Lauricella et al. 2025).

Mechanistically, EOCRC and LOCRC share similar molecular characteristics, characterized by mutations in the *Tp53* and *Kras* genes alongside activation of driving pathways involving WNT, RAS, and PI3K proteins (Tang et al. 2024). However, EOCRC was associated with a higher rate of microsatellite instability (MSI), at 10.2%, compared with 2.2% in LOCRC (Mi et al. 2025; Tang et al. 2024). Additionally, variants in *Fbxw7* and *Rnf43* were more commonly associated with EOCRC (Monge et al. 2025; Tang et al. 2024). In addition to molecular pathology, differences between EOCRC and LOCRC manifest in prognosis, clinical course, and response to therapies. EOCRC was found to be associated with a better prognosis than LOCRC, with a 5‐year survival rate of 54% compared with 32% for LOCRC (Ruiz‐Grajales et al. 2024). However, EOCRC has been associated with more aggressive symptoms, such as rectal bleeding and abdominal pain, than LOCRC (Fritz et al. 2023; Mi et al. 2025). This is further corroborated by findings that EOCRC is associated with higher histopathological grades than LOCRC, likely due to the lack of CRC screening protocols to detect cancer progression early in younger patients (Fritz et al. 2023; Meester et al. 2026). Therapeutic responses also differ between EOCRC and LOCRC. Existing therapeutic options for CRC include radiotherapy, chemotherapy, immunotherapy, adjuvant therapy, and surgical resection (Romero‐Zoghbi et al. 2025). EOCRC was found to be associated with a higher recurrence rate, with a 5‐year cumulative incidence of 29% compared to 21% for LOCRC (Carbone et al. 2025; Nors et al. 2024). Patients diagnosed with EOCRC were more likely to receive a more intensive chemotherapy regimen than LOCRC because of higher patient tolerance to treatment (Carbone et al. 2025; Lin et al. 2025). Given the downward trend in the affected age group and the rapidly evolving molecular pathology of EOCRC, there is an urgent need to better understand its genetic landscape and identify new molecular targets for pharmacological intervention.

Systems genetics is a multi‐omics approach that integrates genetics, genomics, and systems biology to investigate complex interactions between genetic factors and phenotypic traits in the context of disease (Kim, Martin, et al. 2023). Unlike traditional genetic sequencing techniques, like RNA sequencing or quantitative real‐time polymerase chain reaction (qRT‐PCR), systems genetics is equipped with millions of transcript data as well as phenotypic data that were acquired thoroughly at many levels, from the mRNA level, proliferative index, and many more (Mulligan et al. 2017; Wang et al. 2015). The transcript data can be used to perform transcriptional correlation matrix analysis, which enables quantification of correlation coefficient (*R*‐value) among genes of interest (Huang et al. 2024; Kim, Kulkarni, et al. 2023). Additionally, protein–protein interaction (PPI) network analysis can be conducted to investigate key proteins and their activities modulated by genes of interest (Castellanos‐Girouard et al. 2026; Wimalagunasekara et al. 2023). Functional annotation analyses can also be used with Kyoto Encyclopedia of Genes and Genomes (KEGG), Reactome, or Gene Ontology databases to determine the functional roles of genes of interest within a cell (Elizarraras et al. 2024; Muley 2025). Drug‐gene interaction analysis can be performed to identify existing therapeutics that may be repurposed as treatments capable of interrupting the gene network that drives cancer development (Anghel et al. 2024; Qahwaji et al. 2024). Another powerful tool in systems genetics is spatial multiomics, which enables multidimensional analysis of biological tissues by integrating molecular expression profiles with spatial positions. This provides additional context for tumoral heterogeneity and microenvironmental interactions in the native spatial context of a cancer cell (Li, Zhang, et al. 2025). Spatial multiomics can further reveal the nature of immunotherapy resistance by specific cell types and precise spatial locations. This not only unveils new insights into biological mechanisms but also directs precision tumor immunotherapy through “geographical mapping” of the patient tumor microenvironment (Walsh and Quail 2023). Together, systems genetics enables investigators to conduct comprehensive analysis of a gene of interest through the lens of the central dogma of biology.

In this study, our team conducted a literature review demonstrating recent examples of systems genetics applications in EOCRC research. Following a discussion of findings from the literature review, our team developed a workflow for conducting basic systems genetics analysis that is suitable for investigators at all levels. This is accompanied by a practical application investigating a gene implicated in the pathogenesis of EOCRC. Although this investigation primarily focuses on the molecular pathology of EOCRC, the proposed workflow may be applicable to other research interests and disease contexts. The manuscript is divided into three distinct components: a narrative review of the current literature of systems genetics application in EOCRC, a step‐by‐step educational workflow for conducting and interpreting basic systems genetics analysis based on normal colonic tissues, and a discussion of current advances in systems genetics to address limitations of generalizability and context‐dependency. The results generated from the tutorial represent the authors' original application of publicly available tools and data and should be interpreted as hypothesis‐generating rather than as direct evidence of EOCRC molecular pathology.

## 2 Narrative Review: Systems Genetics Applications in EOCRC Research

With advances in bioinformatics in recent years, systems genetics techniques have been used to improve our understanding of cancer biology. With its rising incidence among younger populations, EOCRC has been no exception to scrutiny through systems genetics analysis. Recent systems genetics studies from 2024 to 2026 enabled the identification of novel molecular signatures, mapping of a network of co‐regulatory genes, and even potential new therapeutic options for EOCRC. Herein, we report recent literature that applies transcriptional correlation, PPI network, functional annotation, and drug‐gene interaction analyses to better understand the molecular landscape of EOCRC.

Transcriptional correlation analysis can be used to identify gene co‐regulation networks and to elucidate correlations between gene expression and phenotypic traits, such as age and tumor‐node‐metastasis (TNM) staging (Huang et al. 2024; Kim, Kulkarni, et al. 2023). Rather than generating new RNA‐sequencing data, investigators can use existing systems genetics databases to perform correlation analyses (Kel et al. 2016; Mulligan et al. 2017). In 2024, Marx et al. conducted a Pearson correlation analysis between a list of 2291 differentially expressed genes (DEGs) from The Cancer Genome Atlas—Colon Adenocarcinoma project (TCGA‐COAD) and patients' age (Marx et al. 2024; Chen et al. 2020). The team identified 8 age‐correlated genes—*Aldob, Fbxl16, Il1rn, Msln, Rac3, Slc38a11, Wbscr27*, and *Wnt11*. In another study conducted by Zhou et al. (2025), the team performed Spearman correlation analysis between a list of 330 DEGs acquired from TCGA‐COAD and 1852 differentially methylated CpG islands (DMCs) (Zhou et al. 2025). The team found 6 genes that were negatively correlated with methylation events capable of gene silencing within the EOCRC landscape—*Piwil1, Qrfrr, Gata4, Chst8, Msfa1*, and *Wnt3a*. Another study by Meng et al. (2026) conducted a Pearson correlation analysis between the transcript level of *Etv4* and the transcript levels of mRNA corresponding to 22 types of tumor‐infiltrating immune cells, obtained from TCGA‐COAD (Meng et al. 2026). The team discovered positive correlations with activated CD4 memory T cells and M0 Macrophages, and negative correlations with resting Mast cells and M2 Macrophages.

PPI network analysis can be utilized to investigate protein interactions and signaling networks modulated by genes of interest (Castellanos‐Girouard et al. 2026; Wimalagunasekara et al. 2023). This enables the investigators to predict the functional consequences of polymorphism in a gene of interest. In the case of EOCRC, Wang et al. (2026) conducted Next‐Generation Sequencing (NGS) on 90 EOCRC and 54 LOCRC patients to identify 517 genes frequently mutated in EOCRC patients (Ponvilawan et al. 2025; Szklarczyk et al. 2025). The identified genes were mapped onto the PPI network using the STRING database, and the team discovered four proteins that were enriched in EOCRC: ARID1A, KMT2B, FBXW7, and NOTCH1. The four proteins are involved in chromatin remodeling and the Notch signaling pathway, which may be abnormal in EOCRC. In another study conducted by Nguyen et al. (2025), the team acquired transcript data from 15 colorectal cancer datasets via the cBioPortal database and identified enrichment of 89 genes that were consistently overexpressed in patients with EOCRC (De Bruijn et al. 2023; Nguyen et al. 2025). The team mapped the enriched genes onto a PPI network using the STRING database and identified four proteins enriched in EOCRC patients: GMNN, KPNA2, MYC, and PRDX4. The four proteins are associated with modulation of the mTORC1 signaling pathway, glycolysis, the G2‐M checkpoint, and E2F transcription factors. Another study by Li, Wang, et al. (2025) used targeted deep sequencing of 6530 tissue samples from Chinese patients with metastatic CRC, which were further stratified into EOCRC and LOCRC based on age (Li, Wang, et al. 2025). The team identified 101 driver genes in the EOCRC group and generated a PPI network using the STRING database. The PPI network revealed that MTOR, CHEK2, and QKI were the key driver proteins for LOCRC but not in EOCRC.

Functional annotation is a tool that analyzes a gene network and reveals signaling pathways, biological processes, molecular functions, and other functional features the network regulates (Elizarraras et al. 2024; Muley 2025). Recall the study conducted by Marx et al. (2024) (Gene Ontology 2026). After identifying the 8 age‐correlated genes from transcriptional correlation analysis, the team conducted functional annotation analyses of 48 genes uniquely expressed in EOCRC and of 33 genes exhibiting alternative splicing events uniquely in EOCRC via the Gene Ontology database. The team discovered enrichment of immune‐related terms, T‐cell regulation, cell–cell adhesion, and Wnt signaling pathways, suggesting changes in the immune microenvironment and impaired cellular communication in EOCRC. In another study conducted by Wu et al. (2025), the team identified DEGs from 617 EOCRC samples using TCGA‐COAD and performed functional annotation analysis using the KEGG database (Wu et al. 2025). The team identified enrichment of 11 KEGG pathways, with the strongest enrichment found in the PI3K‐Akt/lipolysis pathway and fatty acid transport. The team also discovered that one of the key proteins identified, FABP4, displayed a positive correlation with the immunosuppressive tumor microenvironment. Another study conducted by Hasan et al. (2026) performed 16S rRNA sequencing on 50 patient‐matched EOCRC and adjacent normal tissues to analyze the microbiota composition of fecal samples from EOCRC (Hasan et al. 2026; Chen et al. 2024). The team specifically identified *L. buccalis, F. alocis*, and *C. aerophile*, which were associated with higher mortality risk and advanced TNM stages. Functional annotation analysis of the identified gut microbiota species was conducted using the KEGG Orthology database and revealed enrichment in the MAPK signaling, styrene, and aminobenzoate degradation pathways. The study further suggests that identifying bacterial species in fecal samples may serve as microbial biomarkers for predicting prognosis in EOCRC patients. Pharmacogenomic analysis is a powerful tool that identifies small molecules that can be repurposed to alter signaling and function of gene networks in cancer (Anghel et al. 2024; Qahwaji et al. 2024). Because pharmacogenomics, or drug‐gene interaction analysis, is one of the newer systems genetics techniques, its application specifically in EOCRC has not been published extensively as of April 2026. However, pharmacogenomics and drug‐gene interaction analysis have been applied to CRC across all ages to identify novel therapeutic agents capable of altering gene networks. In 2025, Liu et al. conducted GWAS (genome‐wide association studies) on 637,693 CRC and healthy patient samples, combined with an analysis of 617 CRC samples from TCGA‐COAD, to identify genetic variants associated with CRC in the Chinese population (Liu et al. 2025; Yoo et al. 2015). The team identified 5 key genes that were negatively correlated with overall survival, relapse‐free survival, and post‐progression survival: *Cdkn2b, Boc, Scg5, Pygl*, and *Metrnl*. The team then conducted pharmacogenomics and virtual screening via DSigB database and AutoDock Vina to identify 10 small molecules capable of altering tumor metabolism through inhibition of PYGL protein: CID 1917711, CID 12305574, CID 12304855, CID 12636254, CID 12636252, CID 12636253, CID 21544425, CID 21544424, CID 21544423, and CID 12636250. Another study by Lei et al. (2025) obtained transcript data for CRC from multiple datasets via GEO and TCGA‐COAD (Lei et al. 2025; Cannon et al. 2024). The team then mapped DEGs and conducted Drug‐Gene interaction analysis via the DGIdb platform. The drug‐gene interaction analysis identified 4 drugs that could alter the cancer signaling pathway by interfering with a central genetic driver, CCNA2: seliciclib, suramin, tamoxifen, and genistein. In another study conducted by Hong et al. (2025), the team conducted cis‐ expression quantitative trait loci (cis‐eQTL) and Mendelian randomization analysis on 4497 druggable genes for CRC acquired from DGIdb and the human druggable gene platform (Hong et al. 2025; Buniello et al. 2025; Knox et al. 2024). The team identified 47 genes associated with CRC development, generated a PPI network, and assessed drug‐gene interactions using the DGIdb, OpenTargets, and DrugBank databases. The team identified 46 drugs capable of disrupting the PPI network by targeting 6 key hub proteins to modulate ECM remodeling, tumor proliferation, immune responses, mitotic progression, and DNA repair: LAMC1 via investigational Laminin‐targeted agents, TFRC via gallium maltolate and ferristatin II, TNFSF14 via pateclizumab, TSSK6 via experimental small‐molecule inhibitors, PLK1 via volasertib, onvansertib, and rigosertib, as well as TYMS via 5‐fluorouracil, raltitrexed, capecitabine, and pemetrexed. It is important to note that the majority of the pharmacogenomics studies summarized above were conducted in pan‐CRC cohorts without age stratification, a limitation that future EOCRC‐focused pharmacogenomics or drug‐gene interaction studies should prioritize addressing.

### 2.1 Tutorial Protocol: Systems Genetics Workflow With Practical Application Investigating *Pik3ca* Gene in Healthy Colonic Tissues

Systems genetics analysis is a multi‐omics tool that can appear challenging to beginners. In this section, our team devised a workflow for conducting basic systems genetics analyzes applicable for learners of all levels, specifically focusing on transcriptional correlation matrix, PPI network, functional annotation, and drug‐gene interaction analyzes. In the workflow, our team investigated the *Pik3ca* gene as a practical application, as the gene has been identified as a key hub protein within the PPI network of studies, and other studies identified enrichment of the PI3K‐AKT pathway in functional annotation analysis of EOCRC (Leiphrakpam and Are 2024; Vital 2025; Yasin et al. 2024). Furthermore, the *Pik3ca* gene has clear identifiers across GeneNetwork, STRING, DrugBank, and other systems genetics databases, which simplifies the workflow for learners. Lastly, small molecules targeting the PI3K/AKT/mTOR pathway are already in development for experimental and clinical studies, enabling drug‐gene interaction studies to identify known compounds (Davoodi‐Moghaddam et al. 2023). We emphasize that this choice reflects a teaching‐and‐methods‐illustration rationale rather than a claim that *Pik3ca* is the optimal biomarker for EOCRC. The workflow presented herein has been verified by three independent medical students who successfully replicated the results. Readers are highly encouraged to access the Supplemental PDF File for visual aids of each step (File S1).

Step 1. Data Acquisition:

In this demonstration, Gene Network 2.0 (https://genenetwork.org/; accessed April 7, 2026) was used to access transcript data from 172 sigmoid colon tissues (Mulligan et al. 2017; Churakov et al. 2025). Users can generate a free account by entering their email address. It is important to note that the dataset contains normal sigmoid colon tissue in the general GTEx population rather than from EOCRC tumor tissue. This approach was deliberately chosen due to the inherent limitations of GeneNetwork 2.0, which currently lacks a dedicated, pre‐processed EOCRC‐specific tumor dataset. While comprehensive tumor‐specific databases, such as The Cancer Genome Atlas Colon Adenocarcinoma (TCGA‐COAD), exist, using them requires complex, multi‐step bioinformatics pipelines, including multivariate Cox regression and non‐negative matrix factorization, that exceed the scope of a foundational tutorial for beginners (Tomczak et al. 2015). By using the GTEx database, this workflow establishes a healthy baseline regulatory network, a standard oncology bioinformatics practice for differentiating true malignant transcriptomic reorganization from normal physiological states. Accordingly, the gene co‐expression network constructed in the steps below should be interpreted as a hypothesis‐generating model of baseline colonic gene regulation, not as direct evidence of EOCRC‐specific tumor biology. Findings from this workflow are intended to illustrate the systems‐genetics method and to generate testable hypotheses for follow‐up in EOCRC tumor cohorts, rather than to stand as conclusions about EOCRC molecular pathology in their own right.

- For the Species field, select “Human.”
- For the Group field, select “GTEx v5 All Tissues.”
- For the Type field, select “Colon – Sigmoid mRNA”
  - For this demonstration, the sigmoid colon was chosen because the incidence of EOCRC has been associated with the left side of the colon more than with the right (Siegel et al. 2026).
- For the Dataset field, select “GTEXv5 Human Colon‐Sigmoid RefSeq (Sep15) RPKM log 2.”
- For the Get Any field, type in the gene of interest: *Pik3ca*.
- Click the Search button.
- On the search result page, click the Record ID for *Pik3ca*: ENSG00000121879.3.
- On the Trait and Analysis page, verify the location of the *Pik3ca* gene on chromosome 3: Chr 3@178.865902 Mb on the plus strand.
- On the Trait and Analysis page, save the gene by clicking the green “Add” button.
- On the pop‐up Define or Add to Collection page, create a new collection by typing in the preferred name: “PIK3CA Systems Genetics” for this exercise. Click “Create Collection” to complete saving.

Step 2. Transcriptional correlation analysis:

Correlation Matrix Analysis: This tool enables users to conduct the standard Pearson or Spearman correlation analysis of mRNA expression between two genes. Unlike partial correlation analysis, this tool calculates a correlation coefficient for a narrow list of genes.

**FIGURE 1:**
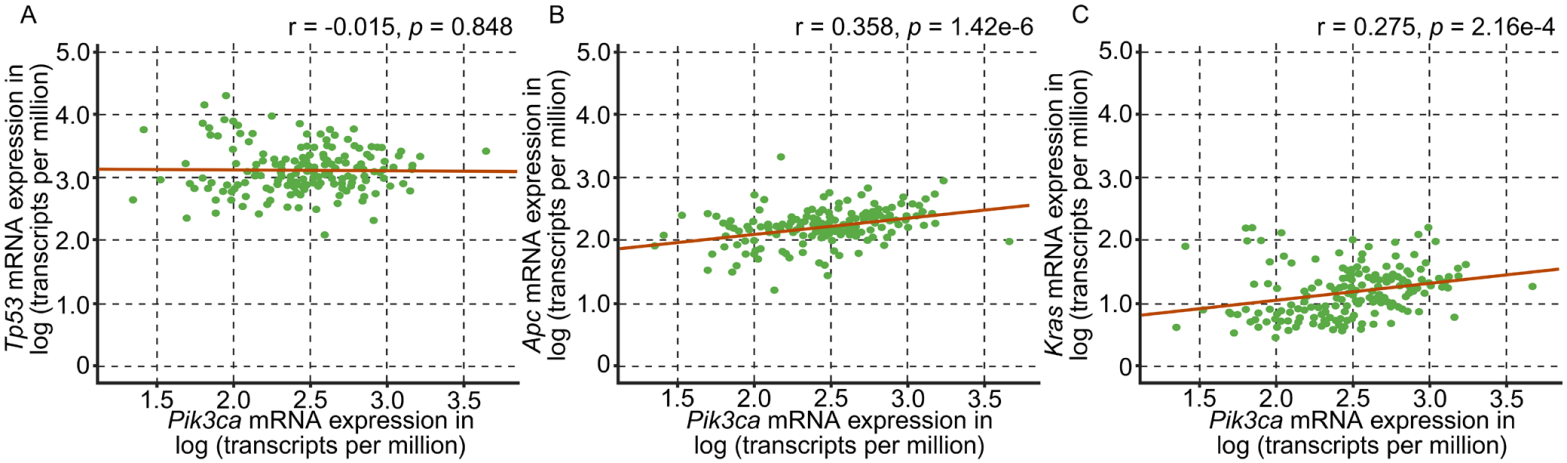
Pearson correlation analyzes of *Pik3ca* mRNA expression levels against (A) *Tp53*, (B) *Apc*, and (C) *Kras* in human colonic tissues. Figure was generated using BioRender.

- For this demonstration, repeat Step1 a‐I for the following genes canonically associated with the molecular pathology of CRC: *Tp53, Apc*, and *Kras*.
  - For reference, Record IDs for *Tp53, Apc*, and *Kras* are ENSG00000141510, ENSG00000134982, and ENSG00000133703.7, respectively.
- For each gene, on the pop‐up Define or Add to Collection page, click “Add to an existing collection” and select: “PIK3CA Systems Genetics” for this exercise. Click “Add” to complete saving.
- Once all 3 genes have been added to your collection, open the collection and click “Select All” to highlight all four genes. Then click the blue “Correlations” box to conduct Correlation Matrix analysis. Click the correlation values to visualize the correlation plot for each pair of genes (Figure 1).
  - The Pearson correlation method is selected over the Spearman correlation method because the dataset consists of continuous expression values and has a relatively large sample size (*n* = 172) per the central limit theorem (Kwak and Kim 2017). Additionally, the approximately normal distribution of *Pik3ca* transcript data was confirmed by Shapiro–Wilk testing (W = 0.992, *p* = 0.430) in R (Chan 2018). Hence, the Pearson correlation method was considered an appropriate default for this hypothesis‐generating scan. However, had the dataset contained a smaller sample size (*n* < 30) with non‐normal data, the Spearman method would be more appropriate (Mulligan et al. 2017).

Partial Correlation Analysis: Unlike correlation matrix analysis, this tool enables a broader investigation of genes and identifies 100 to 20,000 genes that are co‐expressed with a gene of interest.

d Navigate back to the Trait and Analysis page for the *Pik3ca* gene and select the Calculate Correlations tab.
e For the Method field, select “Sample R.”
f For the Database field, select “GTEXv5 Human Colon‐Sigmoid RefSeq (Sep15) RPKM log 2.”
g For the Limit field, select “Top 1000” for this exercise.
  - Note that this value ranges from top 100 to top 20,000. The more genes screened, the longer the computational time. For this exercise, a top‐1000 size is selected, as it is often considered a statistical “sweet spot” that balances statistical power and stability against practical constraints of computation and data collection (Bujang 2024).
h For the Type field, select “Pearson.”
  - The Pearson correlation method is selected over the Spearman correlation method for this hypothesis‐generating scan because of a relatively large sample size (*N* = 172) per the central limit theorem, as well as confirmation of normality of the transcript data for our key gene, *Pik3ca*, through Shapiro–Wilk testing.
i For the Location type field, select “Gene.” Leave all the other fields at their default value.
j Click the green “Compute” button to initiate partial correlation analysis.
k Once the result is generated, select all. Then, save the gene table to a new collection (Refer to STEP1i‐j) and name it “PIK3CA Partial Correlation Analysis.”
l Select all once again and click the white “Download” button to save as a CSV file (Table S1).

Step 3. Functional annotation analysis

**FIGURE 2:**
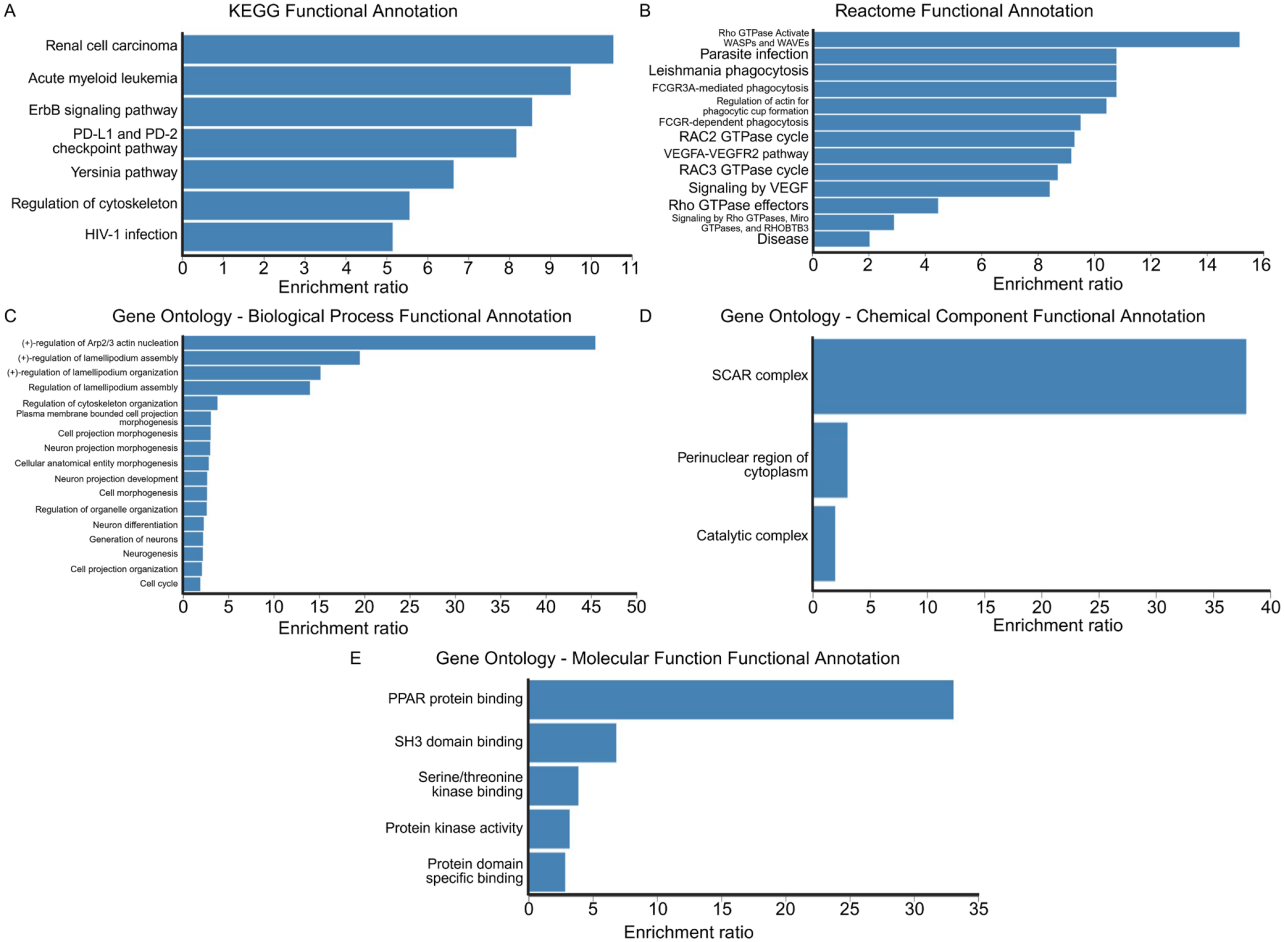
Functional annotation analyzes of top 1000 positively correlated genes with *Pik3ca* transcript levels in human colonic tissues via (A) KEGG, (B) Reactome, (C) Gene Ontology – Biological Processes, (D) Gene Ontology – Chemical Compartment, and (E) Gene Ontology – Molecular function. Figure was generated using BioRender.

- Navigate back to the Collection page containing the results of partial correlation analysis: PIK3CA Partial Correlation Analysis.
- Click the Select All button and then the WebGestalt Button to navigate to the WebGestalt 2024 web portal (https://www.webgestalt.org/; accessed April 13, 2026).
- On the WebGestalt page, select “Over‐Representation Analysis” for the Method of Interest field.
  - Over‐representation analysis is chosen over gene set enrichment or network topology‐based analyzes because of its simplicity, fast computation, and independence from complex parameter tuning, which makes it appropriate for this pedagogical workflow (Chicco and Agapito 2022).
- For the Organism of Interest field, select “ *Homo sapiens* .”
- For the Functional Database fields, add KEGG pathway, Reactome pathway, Gene Ontology Biological Process, Gene Ontology Cellular Component, and Gene Ontology Molecular Function.
  - For this exercise, these five databases are selected, as they are most frequently used in other systems genetics literature discussed in the narrative review. However, investigators can choose other databases suitable for their research, such as Panther, WikiPathways, Mitocarta, and many others (Elizarraras et al. 2024).
- Under the Loaded List tab, the genes from partial correlation analysis should have populated the “Input ID list” field automatically.
- For the ID‐type field, select NCBI Entrez ID.
- Expand the “Advanced parameters” menu and apply the following filter parameters:
  - Redundancy Removal: Check “Weighted set cover (fast).”
    - Gene set databases often contain highly overlapping categories, which can create redundancy in reporting. Hence, the Weighted Set Cover algorithm was selected over affinity propagation or *k*‐medoid, as it can identify the smallest subset of functional categories that covers the maximum number of genes in the input list, effectively prioritizing informative, non‐redundant biological insights (Vivar et al. 2013).
  - Minimum number of analytes for a category: 2; maximum number of analytes for a category: 2000.
    - Setting a minimum threshold of 2 ensures that overly small, statistically fragile categories are excluded, while setting a maximum threshold of 2000 filters out “noisy,” overly broad, or non‐specific gene sets that lack functional specificity (Paton et al. 2024).
  - Multiple Test Adjustment: BH (Benjamini‐Hochberg False Discovery Rate correction).
    - BH FDR correction is chosen over Bonferroni, Holm, or Hommel correction methods, as it balances error control with statistical power. Unlike FWER methods (Bonferroni, Holm, Hommel), which are strictly designed to avoid any false positives, BH allows a small, controlled percentage of false discoveries (FDR) and is considered the standard statistical framework in transcriptomics (Shuken and Mcnerney 2023).
  - Significance Level: FDR < 0.05.
    - An FDR threshold of 0.05 is a widely used and accepted convention in systems genetics to ensure that only 5% of enriched categories are expected to be false positives, providing a balance between sensitivity and specificity (Mulligan et al. 2017; Hasan et al. 2026).
  - Number of categories expected from set cover: 10; number of categories visualized in the report: 40.
    - Selecting 10 as the expected number of categories from set cover instructs the redundancy removal algorithm to converge on a highly concise set of 10 “representative” pathways, preventing the oversight of critical biological signals that can occur when report lists are too large. Selecting 40 categories for visualization provides a comprehensive overview of the broad functional landscape while maintaining the readability and interpretability of the results. There are no “gold standard” values for these parameters, and investigators may choose values appropriate to their research objectives (Elizarraras et al. 2024).
- Click “Submit” and download the results for comprehensive analysis (Figure 2).

Step 4: PPI network analysis.

PPI network generation. In this demonstration, NetworkAnalyst 3.2 was utilized due to its intuitive user‐interaction platform and ease of access to other systems genetics databases (Zhou et al. 2019).

**FIGURE 3:**
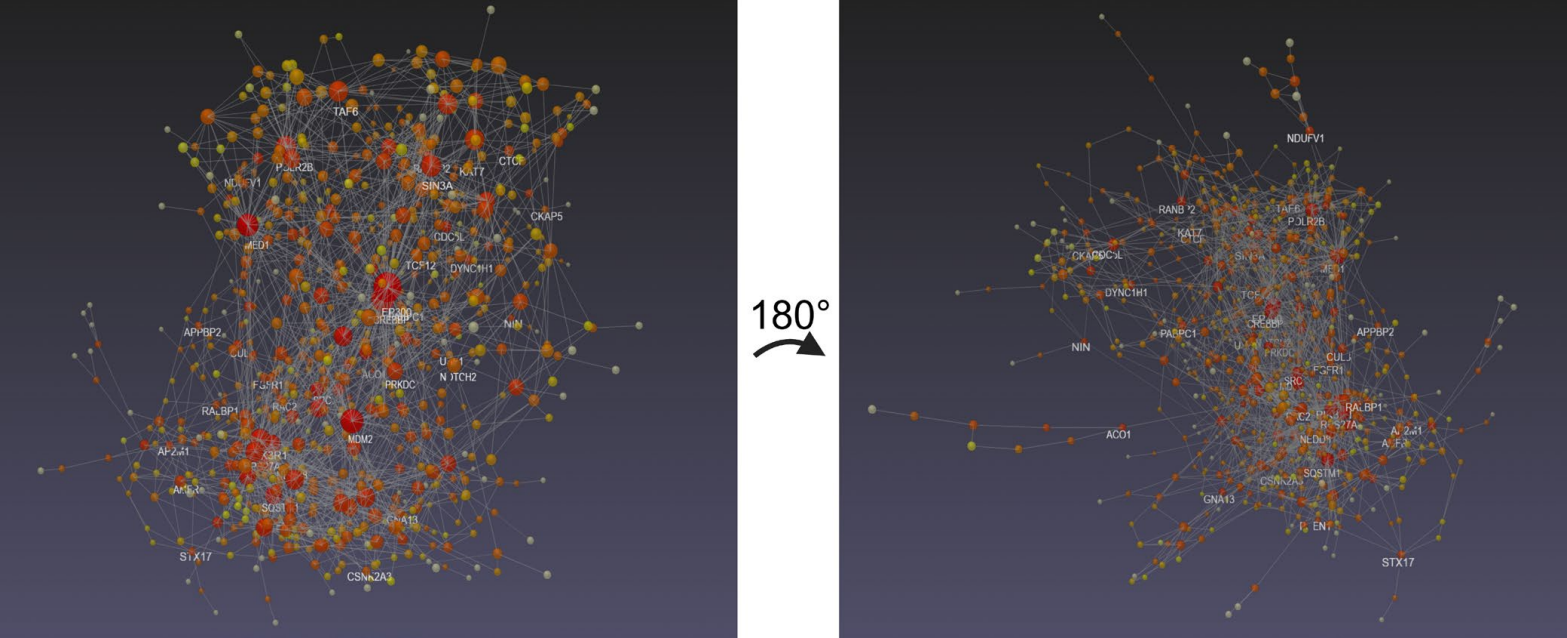
3D PPI network of top 1000 positively correlated genes with *Pik3ca* transcript levels in human colonic tissues.

- Navigate to the NetworkAnalyst 3.2 platform (https://www.networkanalyst.ca/; accessed April 14, 2026) and select “Click here to start.”
- On the Upload Data page, select “ *H. sapiens* (human)” for the Specify Organism field.
- For the Set ID type, select “Official Gene Symbol.”
- Open the CSV file containing genes from partial correlation analysis in Step 2 L. Copy the gene symbols from the CSV file and paste them into the blank input field on the Upload Data page.
- Click the “Upload” button and click “Proceed” on the bottom right.
- On the Analysis Overview page, select “Generic PPI.”
- On the Database selection pop‐up page, select “First order” for the Building Option field.
  - While a “Zero‐order” network maps only the seed genes provided in the input, a “First‐order” network expands the view to include the direct physical and functional binding partners of those seeds. This “First‐order” approach is superior for biological discovery because it captures the local interaction ecology of the target genes (Zhou et al. 2019).
- On the pop‐up page, select “STRING Interactome” as the source of the PPI network database.
  - The STRING Interactome was selected as the primary source for this PPI network analysis because it assigns a confidence score to each interaction by aggregating multiple types of evidence. This multi‐channel integration provides a more expansive, stable, and empirically validated interactome landscape than specialized repositories such as HuRI, the Rolland Interactome, IntAct & IMEx, OmniPath, and InnateDB (Zhou et al. 2019).
- For the Confidence score field, proceed with the default “900” value.
  - Note that confidence score represents the probability that a specific protein–protein interaction is biologically true rather than a false positive. The score ranges from 0 to 1000, with higher scores indicating a greater likelihood that the interaction is a true positive.
  - A score of 900 was selected because it corresponds to the “Highest Confidence” category by integrating multiple high‐stringency evidence channels, including experimental data and curated pathway databases. This ensures that the resulting interactome is built upon the most empirically robust, biologically validated physical binding events (Szklarczyk et al. 2025).
- Select the box for “Experimental evidence” and click the “OK” button. Click the “Proceed” button on the bottom right once the inputs have been processed.
- On the Mapping overview page, select “Minimum Network” on the right side of the page. Once the setting has been processed, click the “Proceed” button.
  - This will generate a subnetwork composed of 845 nodes, 2349 edges, and 382 seeds. Nodes represent the number of proteins in the PPI network. Edges represent the number of interactions between the nodes. Seeds represent the number of genes of interest from your input of 1000 genes that were successfully mapped to the network.
  - For this exercise, “Minimum Network” was selected for the PPI network because this setting kept the number of nodes to less than 1000. Minimum network identifies the smallest possible number of nodes required to connect all the seeds within the PPI network. This effectively filters out non‐essential proteins and avoids the speculative nature of higher‐order (first‐ or second‐order) networks that can dilute the network through indirect connections (Zhou et al. 2019).
- On the result page, select the “3D” button on the left side of the visualization platform to generate the 3D PPI network. Once the 3D PPI network is visualized, generate a GIF animation video of the network by selecting “GIF Animation” under the “Download” tab (Figure 3). Readers are strongly encouraged to view the video analysis of the PPI network to appreciate the 3D nature of the network (Video S1).
  - Generating the GIF Animation file will take roughly 5 to 10 min, depending on the number of nodes and edges of the network.

Functional annotation analysis of PPI network.

m On the result page, identify the “Function Explorer” tab on the right side.
n For the Query field, select “All nodes.”
o For the Database field, select “KEGG.”
  - For this step, users can select from multiple databases: KEGG, Reactome, GO:BP, GO:CC, GO:MF, PANTHER:BP, PANTHER:CC, PANTHER:MF, and Motif. For this exercise, we will focus only on KEGG, Reactome, GO:BP, GO:CC, and GO:MF.
  - Note that NetworkAnalyst 3.2 does not allow users to customize statistical methods and runs with default settings of Benjamini‐Hochberg (BH) for Multiple Test Adjustment Method, FDR < 0.05 for False Discovery Rate, and *p* < 0.05 for significance threshold.
p Once the results table is generated, save it as a CSV file by clicking the “Save” icon next to the “Submit” button.
q Repeat the previous 4 steps for Reactome, GO:BP, GO:CC, and GO:MF as well (Table 1). Download the results for comprehensive analysis (Table S2).

**TABLE 1:** Functional annotation analysis of the PPI network of *Pik3ca* gene. Top 20 significantly enriched terms are listed for KEGG, Reactome, Gene Ontology—Biological Process (GO‐BP), Gene Ontology—Chemical Compartment (GO‐CC), and Gene Ontology—Molecular Function (GO‐MF) analyzes.

	Enrichment ratio	*p*	False discovery rate
KEGG pathways			
Chronic myeloid leukemia	6.67	2.57E‐23	1.32E‐21
Pancreatic cancer	6.59	1.82E‐22	7.22E‐21
Acute myeloid leukemia	6.27	3.24E‐18	5.73E‐17
EGFR tyrosine kinase inhibitor resistance	6.25	1.83E‐21	4.86E‐20
Endometrial cancer	6.21	6.73E‐16	9.30E‐15
ErbB signaling pathway	6.13	3.74E‐22	1.29E‐20
Colorectal cancer	6.05	6.37E‐22	1.84E‐20
Prostate cancer	5.92	1.43E‐23	1.13E‐21
Endocrine resistance	5.86	2.37E‐23	1.32E‐21
Renal cell carcinoma	5.61	1.64E‐15	2.17E‐14
Bladder cancer	5.54	1.64E‐09	8.03E‐09
Prolactin signaling pathway	5.53	2.61E‐15	3.19E‐14
Autophagy—other	5.42	1.90E‐07	7.20E‐07
Mitophagy—animal	5.34	2.01E‐13	1.75E‐12
Adherens junction	5.19	5.47E‐14	5.12E‐13
Shigellosis	5.14	1.69E‐12	1.17E‐11
Autophagy—animal	5.11	2.91E‐23	1.32E‐21
Non‐small cell lung cancer	5.06	2.54E‐12	1.68E‐11
Thyroid cancer	5.06	1.84E‐07	7.18E‐07
Melanoma	5	4.41E‐13	3.60E‐12
Reactome pathways			
mTORC1‐mediated signalling	9.74	1.24E‐07	1.78E‐06
AKT phosphorylates targets in the cytosol	9.26	3.70E‐08	5.79E‐07
SOS‐mediated signalling	8.58	9.67E‐08	1.45E‐06
Signaling by constitutively active EGFR	8.46	8.30E‐10	2.13E‐08
Spry regulation of FGF signaling	8.34	2.65E‐08	4.24E‐07
SHC1 events in EGFR signaling	8.04	2.26E‐07	3.05E‐06
ARMS‐mediated activation	7.5	4.82E‐07	6.09E‐06
Downregulation of SMAD2/3:SMAD4 transcriptional activity	6.98	1.68E‐08	2.80E‐07
Prolonged ERK activation events	6.72	4.64E‐07	5.91E‐06
Frs2‐mediated activation	6.67	1.79E‐06	2.01E‐05
Signalling to RAS	6.44	1.48E‐08	2.48E‐07
SHC1 events in ERBB2 signaling	6.42	5.64E‐08	8.73E‐07
SHC1 events in ERBB4 signaling	6.37	8.27E‐07	1.01E‐05
TAK1 activates NFkB by phosphorylation and activation of IKKs complex	6.1	1.41E‐06	1.65E‐05
Signaling by Hippo	6.1	1.41E‐06	1.65E‐05
Transcriptional activity of SMAD2/SMAD3:SMAD4 heterotrimer	5.92	4.18E‐11	1.36E‐09
Signalling to ERKs	5.78	2.21E‐09	4.13E‐08
SMAD2/SMAD3:SMAD4 heterotrimer regulates transcription	5.75	2.68E‐07	3.51E‐06
Negative regulation of FGFR signaling	5.56	1.52E‐09	3.43E‐08
Downregulation of TGF‐beta receptor signaling	5.45	1.58E‐06	1.82E‐05
GO‐BP			
Epidermal growth factor receptor signaling pathway	5.2	1.68E‐22	4.92E‐21
Intracellular steroid hormone receptor signaling pathway	5.17	1.70E‐15	1.76E‐14
Phosphatidylinositol_mediated signaling	4.66	2.18E‐16	2.71E‐15
Insulin receptor signaling pathway	4.5	1.25E‐19	2.39E‐18
Transcription initiation from RNA polymerase II promoter	4.2	2.98E‐19	5.32E‐18
Histone modification	4.15	3.32E‐26	1.95E‐24
Covalent chromatin modification	4.04	1.77E‐25	8.54E‐24
DNA_dependent transcription, initiation	3.73	2.11E‐17	2.88E‐16
Intracellular receptor mediated signaling pathway	3.53	2.05E‐16	2.58E‐15
Nuclear import	3.52	5.12E‐14	4.04E‐13
Protein import into nucleus	3.49	1.15E‐13	8.83E‐13
Interaction with host	3.41	2.75E‐23	9.80E‐22
Viral reproductive process	3.32	1.14E‐30	1.33E‐28
Response to hypoxia	3.31	4.08E‐13	2.96E‐12
Chromatin modification	3.28	1.20E‐25	6.16E‐24
Nucleocytoplasmic transport	3.2	2.21E‐18	3.55E‐17
Nuclear transport	3.16	3.93E‐18	5.97E‐17
Interphase of mitotic cell cycle	3.01	6.97E‐18	1.02E‐16
Cellular protein catabolic process	2.97	1.63E‐20	3.83E‐19
Transmembrane receptor protein tyrosine kinase signaling pathway	2.95	3.72E‐30	3.82E‐28
GO‐CC			
DNA_directed RNA polymerase II, core complex	8.75	2.91E‐05	9.78E‐05
Transcription factor TFIID complex	6.8	4.21E‐05	0.000138
Apicolateral plasma membrane	6.13	0.0109	0.0244
DNA_directed RNA polymerase II, holoenzyme	5.49	1.58E‐12	1.43E‐11
Ubiquitin ligase complex	5.32	5.06E‐20	6.32E‐19
Replication fork	5.31	5.62E‐07	2.58E‐06
Nuclear replication fork	5.11	0.000317	0.000881
Extrinsic to plasma membrane	4.93	8.55E‐10	5.50E‐09
DNA_directed RNA polymerase complex	4.86	2.93E‐11	2.27E‐10
Nuclear DNA_directed RNA polymerase complex	4.86	2.93E‐11	2.27E‐10
RNA polymerase complex	4.81	3.66E‐11	2.74E‐10
Mediator complex	4.42	0.00035	0.000959
Actin filament	4.4	2.88E‐05	9.78E‐05
Protein serine/threonine phosphatase complex	4.26	9.07E‐05	0.000276
Nuclear chromatin	4.24	1.26E‐12	1.18E‐11
Histone deacetylase complex	4.24	4.22E‐05	0.000138
Lamellipodium	4.19	4.24E‐10	2.89E‐09
Intercalated disc	4.08	0.000282	0.000802
Spliceosomal complex	4.06	9.40E‐12	8.14E‐11
Chromatin	4	5.81E‐22	7.68E‐21
GO‐MF			
Regulation of DNA_dependent transcription, elongation	5.53	2.43E‐06	1.96E‐05
Steroid hormone receptor binding	5.38	3.14E‐10	5.08E‐09
Histone acetyltransferase activity	5.36	5.63E‐08	6.24E‐07
Insulin_like growth factor receptor binding	5.31	0.00542	0.0212
Thyroid hormone receptor binding	5.13	0.000107	0.000647
MAP kinase kinase kinase activity	4.91	0.00276	0.0113
SMAD binding	4.65	7.65E‐08	7.83E‐07
MAP kinase activity	4.65	0.00904	0.0325
RNA helicase activity	4.33	0.000877	0.0041
Receptor signaling protein serine/threonine kinase activity	4.29	1.54E‐05	0.000103
RNA_dependent ATPase activity	4.21	0.00548	0.0212
Ubiquitin binding	4.13	4.89E‐06	3.72E‐05
Protein N_terminus binding	4.11	1.13E‐07	1.12E‐06
Receptor signaling protein activity	4.05	3.21E‐09	4.69E‐08
Damaged DNA binding	4.04	0.000135	0.000769
Non_membrane spanning protein tyrosine kinase activity	4.04	0.000135	0.000769
ADP binding	4.04	0.00669	0.0247
Histone methyltransferase activity	4.03	1.43E‐05	9.88E‐05
Protein phosphatase binding	4.02	1.53E‐06	1.35E‐05
Histone deacetylase binding	4	7.21E‐06	5.28E‐05

Step 5: Drug‐gene interaction analysis

**FIGURE 4:**
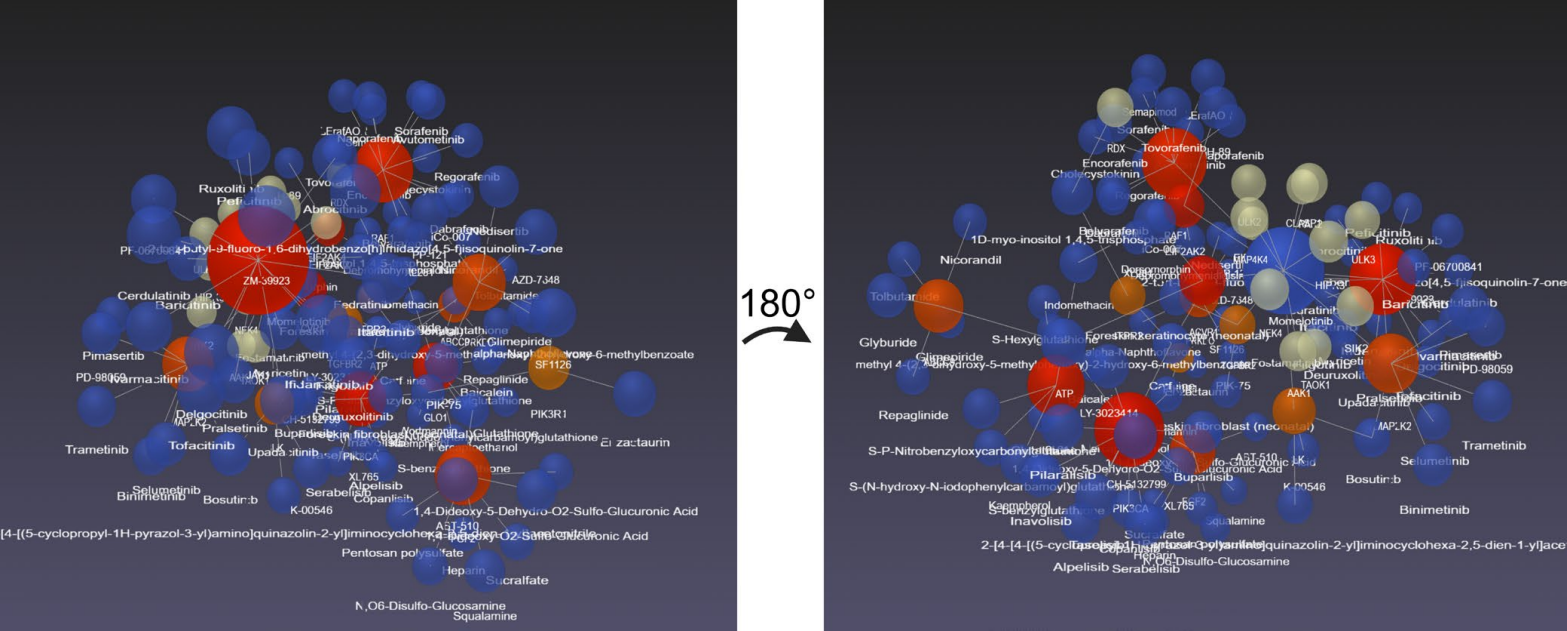
3D drug‐protein interaction network of top 1000 positively correlated genes with *Pik3ca* transcript levels in human colonic tissues.

- On the Network Analyst 3.2 platform, navigate back to the Analysis Overview page.
- Select “Protein‐drug interaction” for analysis type.
- On the pop‐up Database selection, select “DrugBank database” and click OK. Once the inputs have been processed, click the “Proceed” button on the bottom right side of the page.
  - As of July of 2026, there are no other databases available besides DrugBank 5.0. However, as more databases are added, investigators may select those most appropriate to their research objectives.
- On the Mapping overview page, select “First‐order Network” on the right side of the page. Once the setting has been processed, click the “Proceed” button.
  - A first‐order network was selected over the minimum network because the latter yielded too few nodes to capture the broader pharmacological landscape. Selecting the minimum network is excellent for structural pruning in PPI networks with a large number of nodes (*N* > 1000); however, it can inadvertently exclude diverse, secondary pharmacological targets that are critical for understanding drug efficacy and mechanism of action. By contrast, the first‐order network identifies the immediate pharmacological partners of the seed genes, significantly increasing the network's structural density and providing a more comprehensive view of the potential drug‐target interactome (Zhou et al. 2019).
  - This will generate a subnetwork composed of 117 nodes, 119 edges, and 26 seeds.
- On the result page, select the “3D” button on the left side of the visualization platform to generate the 3D network. Once the 3D network is visualized, generate a GIF animation video of the network by selecting “GIF Animation” under the “Download” tab (Figure 4). Readers are strongly encouraged to view the video analysis of the drug‐gene interaction network to appreciate the 3D nature of the network (Video S2).
- Optional: Users can conduct Functional annotation analysis on the 3D protein‐drug interaction network by following the steps of Step 4 m‐q.

### 2.2 Interpretation of the *Pik3ca* Illustrative Workflow Results

Herein, we report the results of the *Pik3ca* workflow. Note that all results are derived from normal sigmoid colon tissues, not from EOCRC; hence the normal colon co‐expression networks may not reflect tumor‐specific, age‐specific, microenvironmental, or treatment‐associated biology in EOCRC. Likewise, lack of experimental validation means the results from the drug‐gene interaction network cannot be used to determine druggability, tumor context, dose, and toxicity of the identified compounds. Accordingly, the findings below represent hypotheses about baseline colonic *Pik3ca* biology that warrant validation in EOCRC‐specific tumor cohorts.

In normal sigmoid colon tissue, *Pik3ca* transcript levels were significantly and positively correlated with both *Apc* (*r* = 0.358, *p* = 1.42 × 10^−6^) and *Kras* (*r* = 0.275, *p* = 2.16 × 10^−4^), while no significant correlation was observed with *Tp53* (Figure 1). The positive co‐expression between *Pik3ca* and *Apc* is notable, as they are classically considered to act through separate mechanisms of PI3K‐AKT–mTOR signaling and Wnt/β‐catenin signaling, respectively (Vital 2025; Brunet Guasch et al. 2025). The positive co‐expression between *Pik3ca and Kras* is consistent with the PI3K–RAS signaling pathway, in which RAS‐GTP recruits and activates class I PI3K isoforms (Ge et al. 2026). The lack of correlation with *Tp53* is biologically coherent, as activation of the *TP53* pathway occurs predominantly in response to genotoxic stress and is not expected to vary in normal tissues (Shim 2025). Among the top 1000 positively correlated genes, several noteworthy *Pik3ca* co‐expressed genes stand out beyond the seed gene itself (Table S1). The strongest non‐seed correlations were observed for *ZRANB1* (*r* = 0.822), *FBXO11* (*r* = 0.798), *MYCBP2* (*r* = 0.794), *EXOC5* (*r* = 0.793), and *WWC2* (*r* = 0.797)—all with correlations exceeding 0.79. Within the canonical PI3K pathway, regulatory subunit *PIK3R1* (*r* = 0.537), downstream effector *AKT3* (*r* = 0.604), scaffold adaptors *IRS1* (*r* = 0.547), and *IRS2* (*r* = 0.517) were co‐expressed with *Pik3ca*. Hippo pathway components, including *SAV1* (*r* = 0.748), *YAP1* (*r* = 0.649), and *STK4* (*r* = 0.489), were also correlated, suggesting possible PI3K–Hippo crosstalk in normal colonic tissue. Epigenetic regulators *EP300* (*r* = 0.631), *CREBBP* (*r* = 0.597), *KMT2A* (*r* = 0.542), and *SETD2* (*r* = 0.526) were also co‐expressed. Taken together, these correlations are consistent with known oncogenic pathway interactions, but the effect sizes are modest, and the relationships are observed in normal—not neoplastic—tissue.

KEGG functional annotation analysis of the top 1000 *Pik3ca*‐correlated genes identified renal cell carcinoma (enrichment ratio ~10.9), acute myeloid leukemia (~8.9), ErbB signaling pathway (~8.2), and PD‐L1/PD‐L2 checkpoint pathway (~8.1) among the top‐ranked terms (Figure 2A). The presence of multiple cancer‐associated KEGG pathways alongside cytoskeletal and immune checkpoint pathways reflects the broad oncogenic and regulatory signaling networks co‐expressed with *Pik3ca* in normal colonic epithelium, and should be interpreted as characterizing the transcriptional neighborhood of *Pik3ca* rather than as evidence of EOCRC‐specific pathway activation. At the Reactome level, the top‐enriched terms were dominated by Rho GTPase effector pathways, including Rho GTPase activation of WASPs and WAVEs, Leishmania phagocytosis, FCGR3A‐mediated phagocytosis, and RAC2/RAC3 GTPase cycles alongside VEGF‐VEGFR2 signaling and Rho GTPase effectors more broadly (Figure 2B). GO‐BP terms were dominated by actin nucleation and lamellipodia assembly and organization (Figure 2C), while GO‐CC featured the SCAR complex (enrichment ratio ~37) (Figure 2D), and GO‐MF identified PPAR protein binding (~32) and SH3 domain binding (~6.5) as the top molecular function terms (Figure 2E). The enrichment of cytoskeletal remodeling and Rho GTPase programs among the top correlates of *Pik3ca* in normal colon is biologically interesting, as it may reflect the co‐regulation of PI3K‐driven proliferative programs with actin‐cytoskeletal activity in intestinal epithelial homeostasis, a state that is frequently disrupted during colorectal carcinogenesis.

The PPI network generated from the top 1000 *Pik3ca*‐correlated genes represented a large network composed of TP53, EP300, CTNNB1 (β‐catenin), CREBBP, SMAD2/SMAD3, MDM2, AKT1/AKT2, SMARCA4, HDAC1, and KMT2A as the most prominent nodes (Figure 3). These nodes cluster into two functionally coherent superclusters: a chromatin remodeling and transcriptional co‐activation supercluster anchored by EP300, CREBBP, SMARCA4, HDAC1, and KMT2A, and a growth factor/oncogenic signaling supercluster anchored by TP53, CTNNB1, AKT1, SMAD2/3, and MDM2. Specifically, TP53 and MDM2 were identified as high‐connectivity nodes, although they were not co‐expressed with *Pik3ca* in the transcriptional correlation analysis. This is a recognized property of STRING‐derived PPI networks, where highly connected proteins accumulate connections through their interaction partners regardless of the seed gene (Wimalagunasekara et al. 2023). Their presence should be interpreted as representing the interaction ecology of the *Pik3ca* co‐expression neighborhood rather than as direct functional evidence of *Pik3ca*–TP53 co‐regulation in EOCRC. By contrast, the chromatin regulatory proteins, including EP300, CREBBP, KMT2A, SMARCA4, and HDAC1, are supported by both their PPI connectivity and co‐expression with *Pik3ca* (EP300 with *r* = 0.631, CREBBP with *r* = 0.597, KMT2A with *r* = 0.542, and SETD2 with *r* = 0.526). CTNNB1 is another hub of particular relevance to EOCRC. Its central position in the Wnt signaling network, together with the co‐expression of Hippo pathway regulators *YAP1* and *SAV1*, which converge on β‐catenin, and the well‐documented Wnt–PI3K crosstalk in CRC, suggest that CTNNB1 represents a biologically plausible node for follow‐up in the EOCRC context (Amerizadeh et al. 2025).

Functional annotation analysis of the PPI network yielded a complementary set of enrichment results across multiple databases (Table 1). At the KEGG level, the highest enrichment ratios were observed for chronic myeloid leukemia (6.67, *p* = 2.57 × 10^−23^), pancreatic cancer (6.59), ErbB signaling pathway (6.13), and colorectal cancer (6.05, *p* = 6.37 × 10^−22^, FDR = 1.84 × 10^−20^). The enrichment of multiple epithelial and hematopathological cancer pathways likely reflects the pan‐oncogenic signaling infrastructure of the *Pik3ca* interaction network rather than CRC‐specific biology. Notably, EGFR tyrosine kinase inhibitor resistance (6.25) and endocrine resistance (5.86) also featured, suggesting that the interaction network includes genes linked to therapeutic resistance mechanisms relevant to gastrointestinal oncology. At the Reactome level, the PPI network annotation yielded results that were more coherent with the canonical signaling role of PIK3CA. The top‐ranked terms, including mTORC1‐mediated signaling (enrichment ratio 9.74, *p* = 1.24 × 10^−7^), AKT phosphorylation of cytosolic targets (9.26), SOS‐mediated signaling (8.58), and signaling by constitutively active EGFR (8.46), delineate the PI3K‐AKT–mTOR‐RAS‐EGFR axis as the dominant signaling core of the *Pik3ca* interaction network. Additional enriched terms were TGF‐β/SMAD signaling (enrichment ratio 6.98) and Hippo signaling (enrichment ratio 6.10), which are consistent with the presence of *IRS1*, *IRS2*, *AKT3*, and *PIK3R1* among the top‐correlated genes (Table S1).

The GO‐BP results revealed enrichment of EGFR signaling (enrichment ratio 5.20) and insulin receptor signaling (4.50) ranked highest as expected, but the most significantly enriched single GO‐BP term was histone modification (4.15, *p* = 3.32 × 10^−26^, FDR = 1.95 × 10^−24^), followed by covalent chromatin modification (4.04, *p* = 1.77 × 10^−25^) and chromatin modification (3.28, *p* = 1.20 × 10^−25^). These outcomes are reinforced at the GO‐MF level, where histone acetyltransferase activity (enrichment ratio 5.36), histone methyltransferase activity (4.03), and histone deacetylase binding (4.00) all reached significance, and at the GO‐CC level, where histone deacetylase complex (4.24) and nuclear chromatin (4.24) were enriched. The co‐expressed epigenetic regulators driving these signals, including *EP300* (*r* = 0.631), *CREBBP* (*r* = 0.597), *KMT2A* (*r* = 0.542), *SETD2* (*r* = 0.526), *KAT2A*, and *KAT7*, are supported both by their transcriptional co‐expression with *Pik3ca* and by their PPI network connectivity. This convergent evidence across GO ontologies of epigenetic and chromatin‐regulatory enrichment in the *Pik3ca* interaction network is, to our knowledge, an underexplored dimension of *PIK3CA* biology in the colorectal context and may merit dedicated experimental follow‐up. The GO‐MF results further revealed enrichments of steroid hormone receptor binding (5.38) and SMAD binding (4.65), linking the PPI network to nuclear receptor and TGF‐β regulatory functions. Together, these results portray a complex network integrating PI3K/EGFR/mTOR growth factor signaling, epigenetic chromatin regulation, and TGF‐β/Hippo signaling pathways.

The drug‐gene interaction network mapped to DrugBank 5.0 database contained 117 nodes and 119 edges, yielding a structurally organized map of candidate drug agents classifiable into mechanistically coherent groups (Figure 4). The most directly on‐target candidates were the PI3K pathway inhibitors, which target the seed gene and its immediate effectors: alpelisib, copanlisib, buparlisib, taselisib, pilaralisib, serabelisib, and the dual PI3K/BRD4 inhibitor, SF1126. Of these, alpelisib has the strongest existing mechanistic rationale for *PIK3CA*‐mutant CRC, as its clinical benefit in *PIK3CA*‐mutant breast cancer has prompted investigation in *PIK3CA*‐mutant colorectal settings, though regulatory approval in CRC has not been established (Voutsadakis 2026). The network also identified RAF/MEK/ERK axis inhibitors, such as sorafenib, regorafenib, dabrafenib, trametinib, binimetinib, selumetinib, avutometinib, naporafenib, and encorafenib, as interactors of *Pik3ca* co‐expressed kinase nodes. Notably, regorafenib is the only compound with regulatory approval for metastatic CRC, making it a relevant network‐predicted agent (Dhillon 2018). Additionally, the JAK inhibitors, including ruxolitinib, peficitinib, itacitinib, upadacitinib, and tofacitinib, were identified in the network via *JAK1*, one of the top 20 most strongly correlated genes, with a correlation coefficient of 0.751. JAK1 inhibition has shown mixed results in CRC, and current evidence does not support the use of JAK inhibitors as a primary treatment for CRC (Li and Huang 2024). Hence, they appear in this network as hypothesis‐generating targets in the context of EOCRC at this time. Similarly, enzastaurin, a PKCβ/PI3K inhibitor, and pimasertib, a MEK inhibitor, have no established clinical benefit in colorectal disease. A cluster of FGF2‐linked anti‐angiogenic and heparan sulfate‐binding compounds, including ABT‐510, pentosan polysulfate, squalamine, sucralfate, and heparin‐related glucuronic acid derivatives, appears through FGF2, which is a DrugBank‐curated interactor of network nodes as opposed to a direct *Pik3ca* co‐expression target; these should be regarded as low‐priority, database co‐occurrence candidates.

It is important to note that all the results from this workflow should be read strictly as hypothesis‐generating rather than as evidence of clinical efficacy. The network was generated from normal colonic tissues and publicly curated protein–drug interactions. The results cannot be used to extrapolate tumor‐specific target expression levels, pharmacokinetic feasibility at the colonic tumor site, therapeutic window, off‐target toxicity, or drug–drug interactions in EOCRC. Additionally, the absence of age‐stratified or EOCRC‐specific pharmacogenomics data in the underlying databases means that compounds nominated by drug‐gene interactions will require proper validation before any clinical translation can be considered. Notwithstanding these limitations, the workflow successfully recapitulates biologically plausible drug–target relationships, such as PI3K inhibitors for a *Pik3ca*‐seeded network and regorafenib, a clinically established CRC agent, thereby demonstrating its utility as a hypothesis‐generating tool accessible to researchers at all levels.

## 3 Discussion of Current Limitations and Future Outlook of AI‐Enhanced Systems Genetics

While systems genetics analyzes aided in the discovery of novel biomarkers for EOCRC and potential therapeutic avenues, the generalizability of the results is a major limitation. Existing systems genetics databases enable users to investigate a variety of tissues, from healthy colon tissues to metastatic CRC tissues. However, tissue samples often neglect the contribution from environmental factors, such as smoking, dietary conditions, or drug use, which are highly relevant to the pathophysiology of EOCRC (Wang et al. 2026; Li et al. 2023; Brim et al. 2025). For example, while systems genetics findings based on Chinese CRC tissue samples offer new insights into the molecular pathology of EOCRC (Li, Zhang, et al. 2025, Liu et al. 2025), the differences in biopsychosocial factors limit the extrapolation of these results to the United States or other regions of the world. In fact, twin studies in CRC identified a heritability of 40%, suggesting that environmental factors play a significant role alongside genetic differences (Graff et al. 2017). Furthermore, systems genetics databases often acquire transcript data from animal models, such as BXD and LXS recombinant mice (Li et al. 2024; Radcliffe et al. 2020). However, the translatability of results from transgenic mice to human clinical data has not been thoroughly verified.

Fortunately, current advances in bioinformatics tools aim to address issues with the generalizability of systems genetics result, specifically through the application of spatial multiomics and artificial intelligence (AI) models. In 2023, Furuhashi et al. performed spatial transcriptomics analysis of EOCRC tissues and identified fibroblast‐associated protein (FAP)‐positive cancer‐associated fibroblasts at the tumor invasive margin of EOCRC (Furuhashi et al. 2023). The analysis revealed higher mRNA and protein levels of fibroblast growth factor receptor 2 (FGFR2) and PI3K/Akt signaling proteins in these cells compared with neighboring tumor cells. Furthermore, EOCRC patients with higher FAP levels in cancer‐associated fibroblasts had shorter overall survival, providing new spatial context of cancer‐associated fibroblasts in the tumor microenvironment of EOCRC. On the other hand, in 2024, Jayakrishan et al. trained a machine‐learning AI model, DIABLO (Data Integration Analysis for Biomarker Discovery), using metabolomics and 16S rRNA sequencing data from 64 EOCRC and 49 healthy tissues in the Cleveland Clinic Biobanks (Jayakrishnan et al. 2024). The trained AI model integrates metabolomics and 16S rRNA sequencing data to identify molecular biomarkers present in both datasets, whereas traditional methods analyze these datasets separately. The study successfully identified a cluster of *Akkermansia* microbes present only in EOCRC and suggested the use of a fecal microbial assay as a screening biomarker specifically for EOCRC. In another study conducted by Yue et al. (2025), the team trained a combination of two machine learning AI models, Lasso (Least Absolute Shrinkage and Selection Operator) and GBM (Gradient Boosting Machine), on 420 CRC samples acquired from the TCGA‐COAD database (Yue et al. 2025). The team then tested their AI model on multiple CRC transcript datasets to improve the generalizability of the results and identified *Lsm8*, a key gene involved in the metabolism of amino acids and their derivatives, as a high‐risk gene for the development of CRC. The team then validated the gene by confirming in vitro that silencing *Lsm8* prevented CRC cells from migrating. Lastly, another study conducted by Diaz et al. (2026) utilized Large Language Models (LLMs), which have been trained on billions of medical literature and genomic codes, to process CRC transcript data from 2515 patients across multiple datasets (Diaz et al. 2026). The team then fine‐tuned the LLM to consider contextual factors when interpreting the datasets, such as ancestry, race, age, and immunotherapy status. While traditional systems genetics workflows treat contextual factors as independent variables, the team's LLM uses contextual mapping to identify hidden patterns in the dataset without requiring complex lines of code. The team successfully identified *Nf1* and *Rps6ka4* genes that are significantly enriched among EOCRC Hispanic patients who have not undergone FOLFOX treatment. Together, the integration of spatial multiomics and AI in CRC systems genetics research is expected to enhance the speed, generalizability, and context‐dependent analysis.

## 4 Conclusion

EOCRC is becoming increasingly problematic with its rising incidence among young patients at an alarming rate. As numerous factors driving the progression of EOCRC remain elusive, there is an urgent need to study the molecular pathology, diagnostic markers, and novel therapeutics against this debilitating disease. In the past several decades, the scientific community has amassed millions, if not billions, of sequencing data, some of which have been used to better understand the pathophysiology of EOCRC. Yet there is a wealth of data that has not been used to its full potential. Systems genetics is a powerful tool that enables investigation of multiple genes as a collective network rather than independently. Through transcriptional correlation, PPI network, functional annotation, and pharmacogenomics, systems genetics can aid in understanding the evolving molecular pathology of EOCRC. With advances in spatial multiomics and AI research that actively address the issues of generalizability, translatability, and speed, systems genetics is poised to become an essential tool in cancer biology research and will help fuel current efforts to fight this malignancy. By providing protocols and an illustrative example featuring *Pik3ca*, our team envisions a future in which emerging experimental oncology researchers enthusiastically adopt these tools, seamlessly incorporating them into their ongoing research against EOCRC and beyond.

## Author Contributions

**Minjae J. Kim:** conceptualization, investigation, methodology, visualization, writing – original draft. **Vishnutheertha Kulkarni:** investigation, methodology, writing – review and editing. **Sydney L. Carr:** investigation, methodology, writing – review and editing. **Mary‐Louise Manchadi:** writing – review and editing, supervision.

## Funding

This work was supported by Health Science Center, University of Tennessee and University of Queensland (Grant 2010002134).

## Conflicts of Interest

The authors declare no conflicts of interest.

## Supporting information


**File S1:** Visual workflow for conducting basic systems genetics analyzes for learners of all levels, focusing on the transcriptional correlation matrix, PPI network, functional annotation, and drug‐gene interaction analyzes.

**Video S1:** Video analysis of 3D PPI network of top 1000 positively correlated genes with *Pik3ca* transcript levels in human colonic tissues.

**Video S2:** Video analysis of 3D drug‐protein interaction network of top 1000 positively correlated genes with *Pik3ca* transcript levels in human colonic tissues.

**Table S1:** List of top 1000 correlated genes of *Pik3ca* in normal human colonic tissues.

**Table S2:** Full results of functional annotation analysis of the PPI network of *Pik3ca* and its top 1000 correlated genes.

## Data Availability

The data that support the findings of this study are available on request from the corresponding author. The data are not publicly available due to privacy or ethical restrictions.
