# Supplementary material for: Application of Systems Genetics to Investigate Molecular Pathology of Early‐Onset Colorectal Cancer
Source: Chem Biol Drug Des. 2026 Aug 11;108(2):e70379. doi: 10.1111/cbdd.70379 (PMC13460241; doi:10.1111/cbdd.70379)
Supplement: Supplementary file 1 [file CBDD-108-e70379-s002.pdf]

Step 1a-e: Input parameters

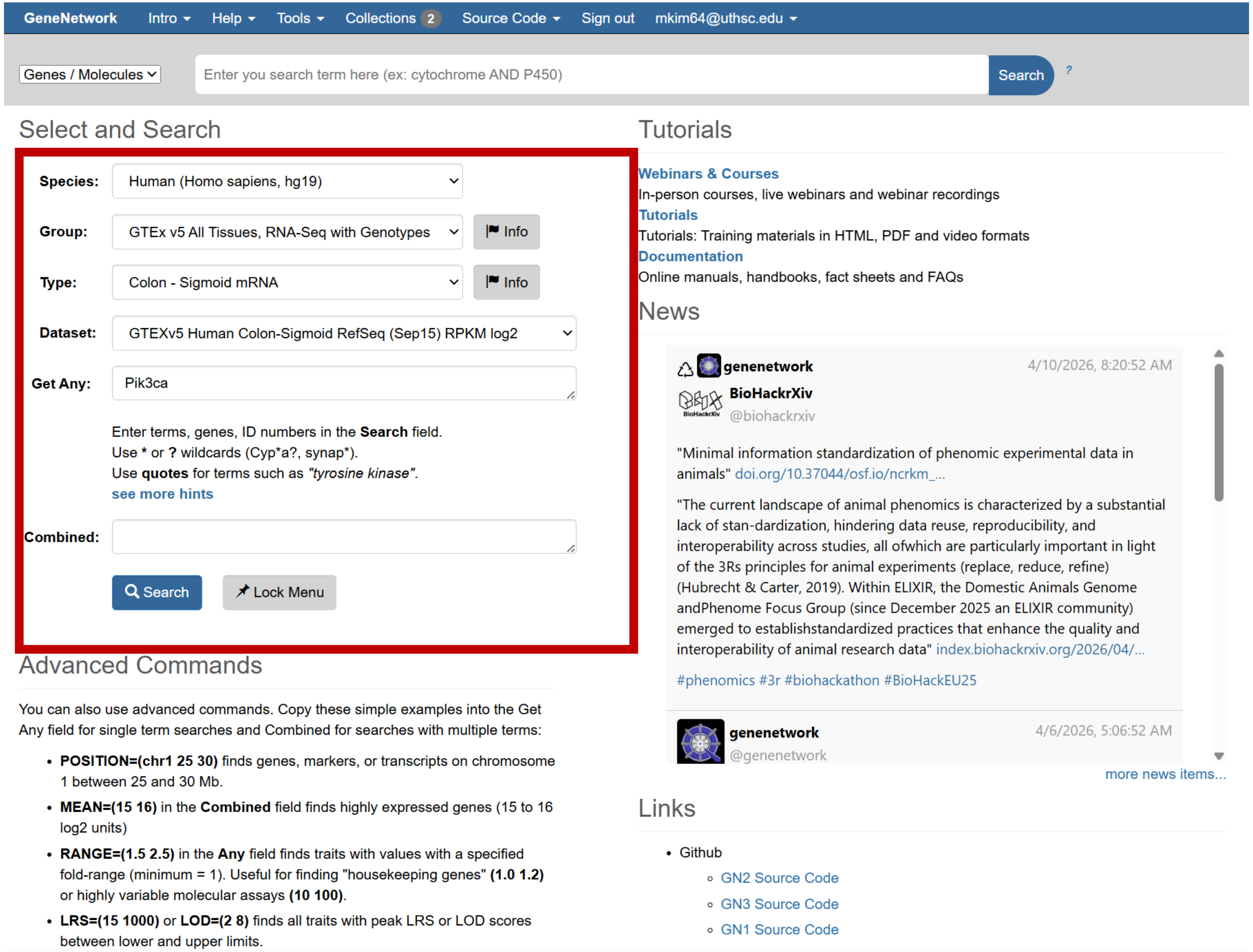

Step 1g: Result page

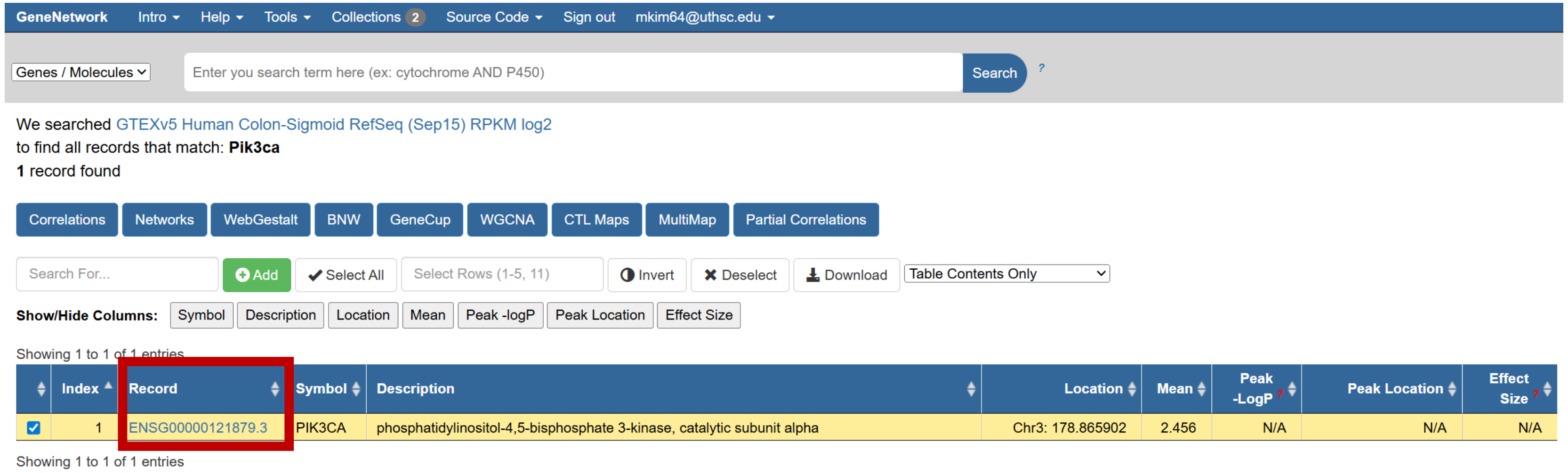

Step 1h-i: Confirm chromosomal location of gene: *Pik3ca* and save the gene using “Add” function

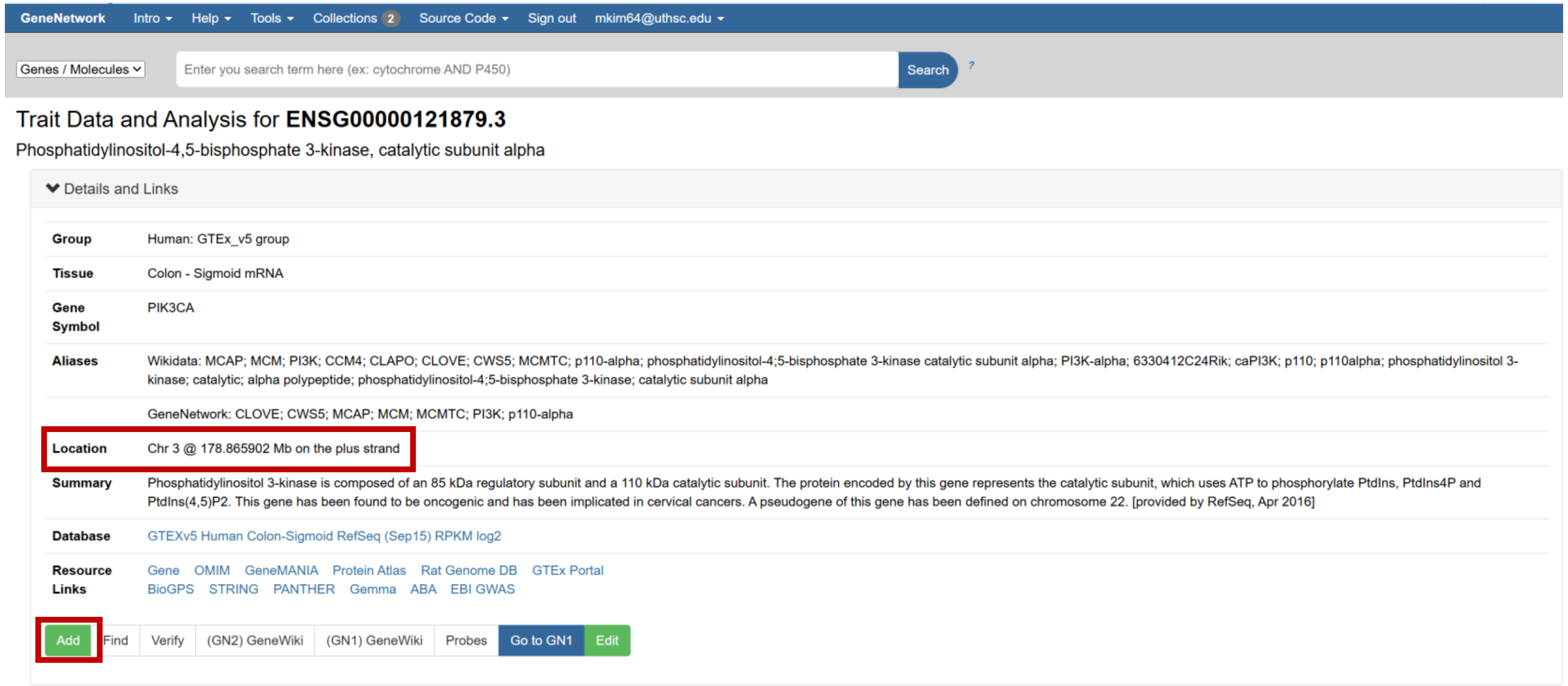

Step 1j: Create new collection and save the gene

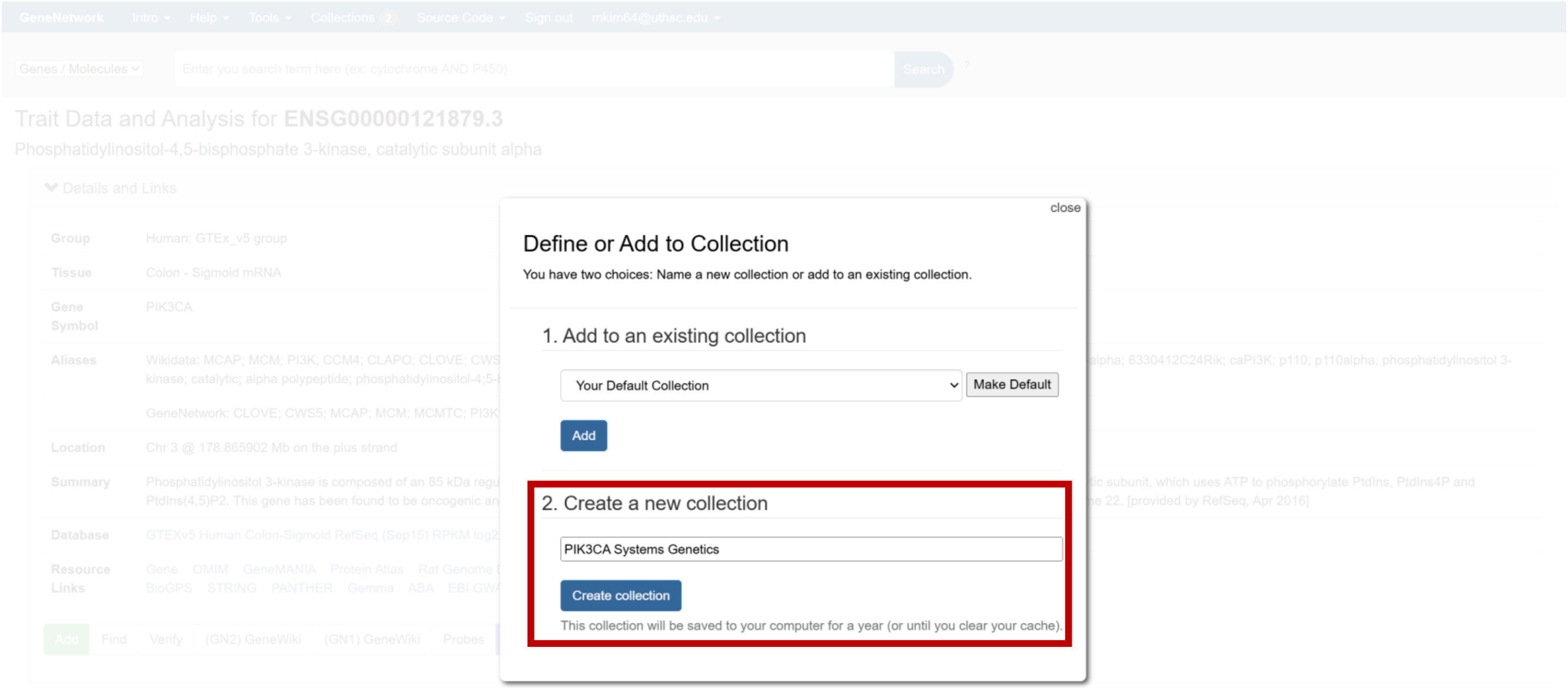

Step 2b: Save genes of interest to existing collection

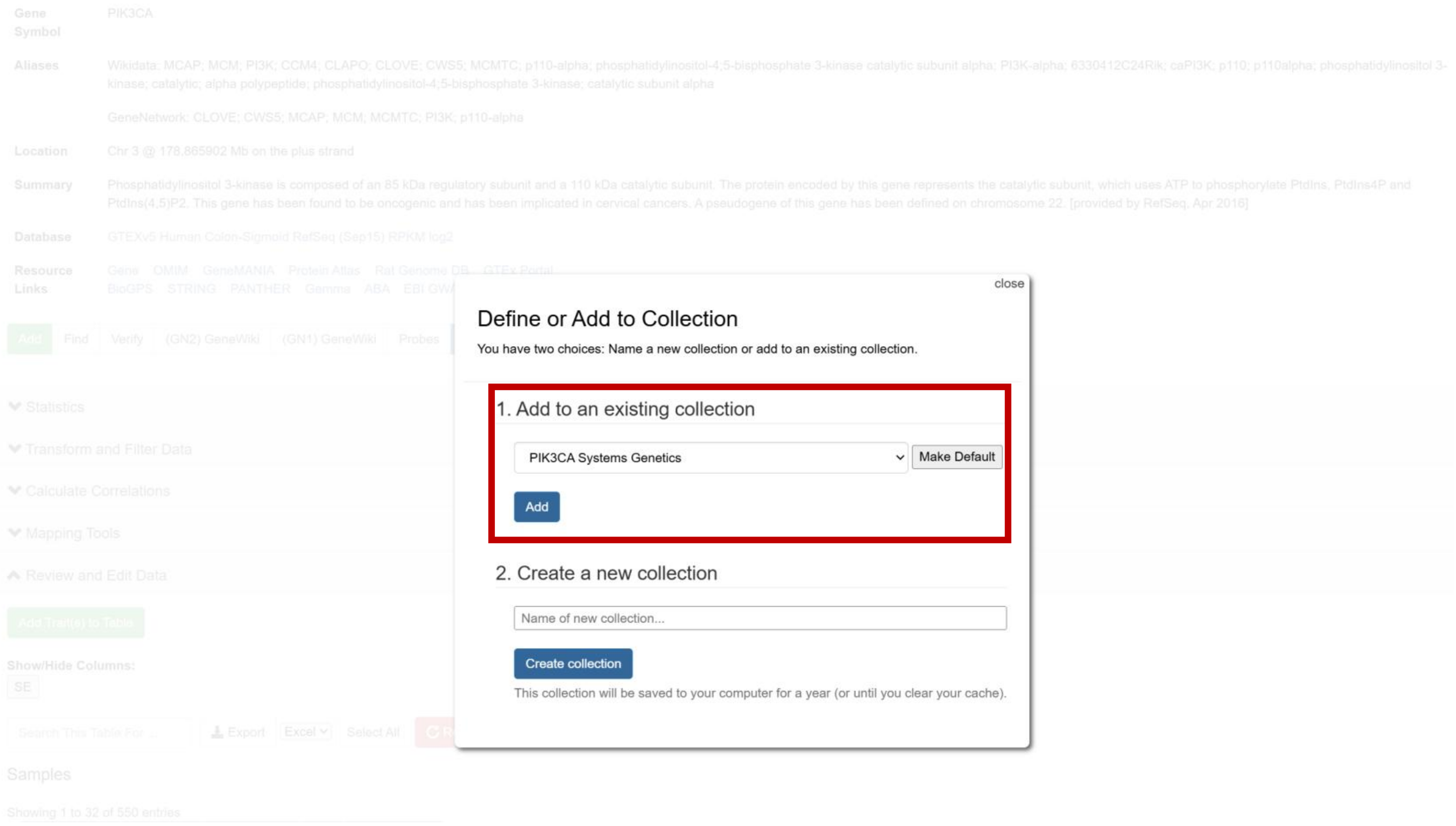

Step 2c: Select all genes of interest and run correlation matrix analysis

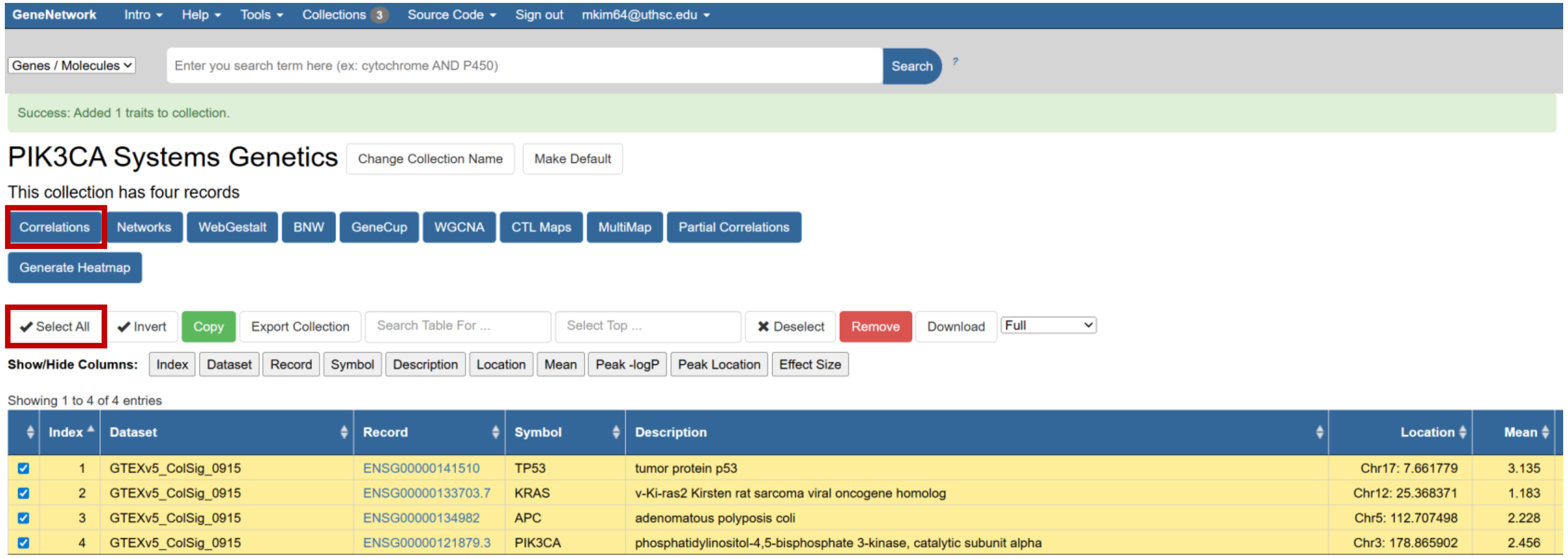

Step 2d: Select “Calculate Correlations” tab for partial correlation analysis

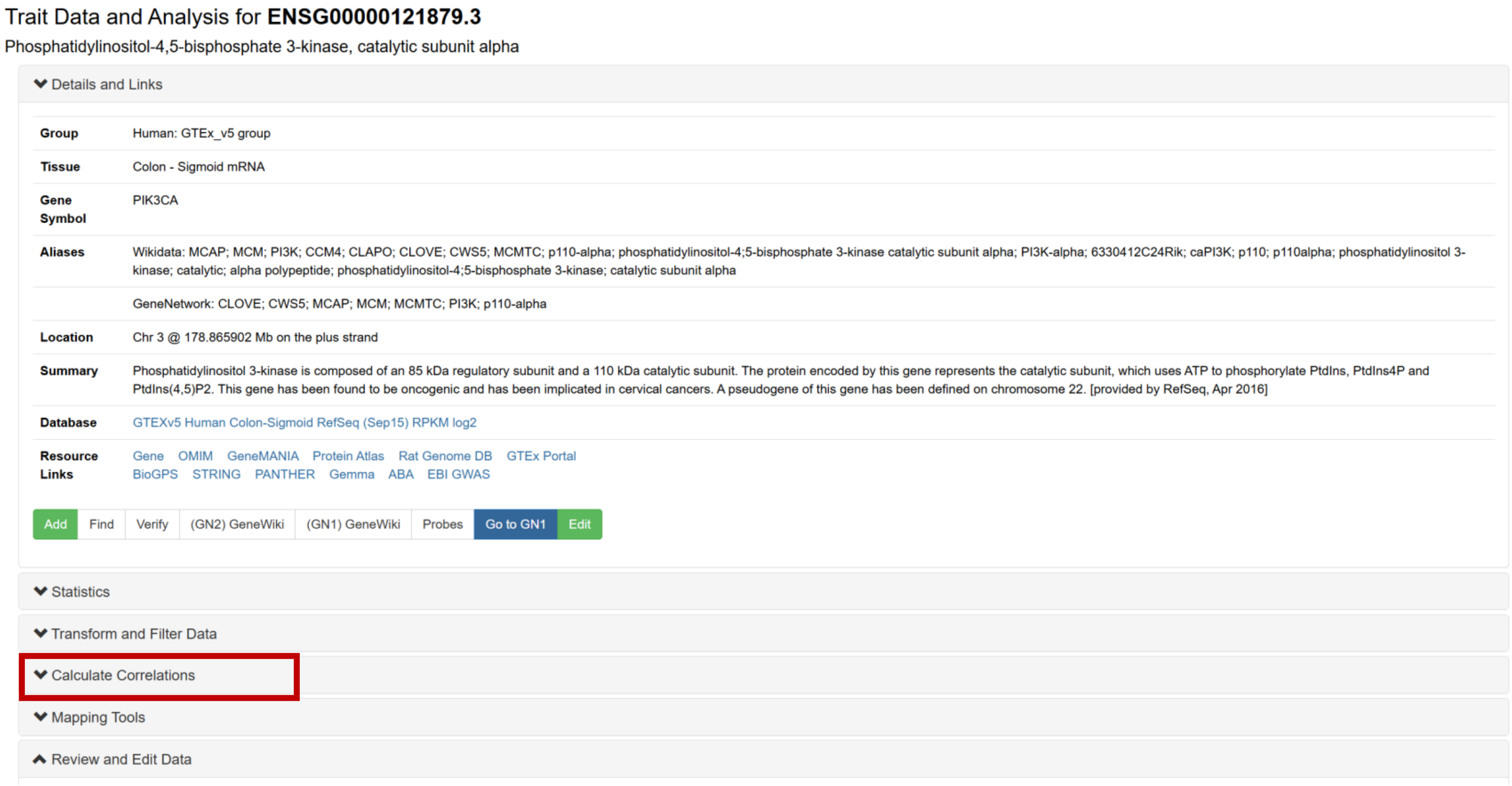

Step 2e-j: Input parameters for partial correlation analysis

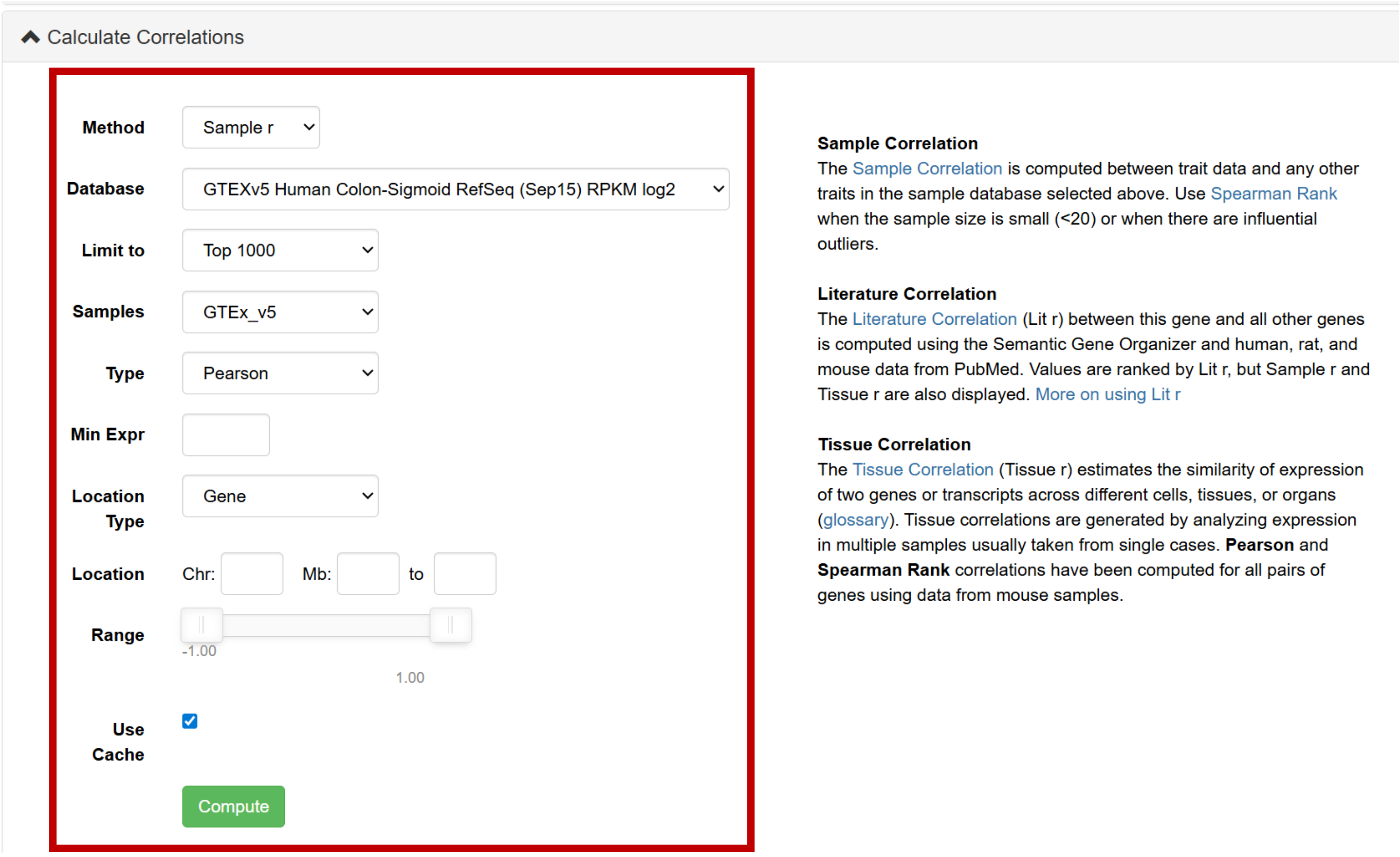

Step 2k: Save top 1,000 correlated genes into new collection

Values of record ENSG00000121879.3 in the GTEXv5 Human Colon-Sigmoid RefSeq (Sep15) RPKM log2 dataset were compared to all records in the GTEXv5 Human Colon-Sigmoid RefSeq (Sep15) RPKM log2 dataset. The top 1000 correlations ranked by the Genetic Correlation (Pearson's r) are displayed. You can resort this list by clicking the headers. Select the Record ID to open the trait data and analysis page.

Correlations | Networks | WebGestalt | BNW | GeneCup | WGCNA | CTL Maps | MultiMap | Partial Correlations

✔ Select All | ◑ Inve | ⊕ Add | Search Table For ... | Select Rows (1-5, 11) | ✖ Deselect | Reset Columns

Download ⓘ

More Options...

Show/Hide Columns:

Symbol | Description | Location | Mean | Peak -logP | Peak Location | Effect Size

Showing 1 to 997 of 997 entries

	Index	Record	Symbol	Description	Location	Mean	Sample R	N	Sample P(R)	Lit R	Tissue R	Tissue P(R)	Peak -LogP	Peak Location	Effect Size
☑	1	ENSG00000121879.3	PIK3CA	phosphatidylinositol-4,5-bisphosphate 3-kinase, catalytic subunit alpha	Chr3: 178.865902	2.456	1.000	172	0.00e+00	--	1.000	0.000e+00	N/A	N/A	N/A
☑	2	ENSG00000249456.1	RP11-298J20.4	pseudogene RP11-298J20.4	Chr10: 126.605712	3.011	0.833	172	1.65e-45	--	--	--	N/A	N/A	N/A
☑	3	ENSG00000019995.6	ZRANB1	zinc finger, RAN-binding domain containing 1	Chr10: 126.630692	3.708	0.822	172	1.94e-43	--	-0.231	2.569e-01	N/A	N/A	N/A
☑	4	ENSG00000236051	MYCBP2-AS1	MYCBP2 antisense RNA 1	Chr13: 77.075514	2.021	0.811	172	2.23e-41	--	--	--	N/A	N/A	N/A
☑	5	ENSG00000226121.1	AC009487.6	long intergenic non-coding RNA AC009487.6	Chr2: 162.357396	1.048	0.802	172	6.45e-40	--	--	--	N/A	N/A	N/A
☑	6	ENSG00000138081.15	FBXO11	F-box protein 11	Chr2: 48.016455	3.289	0.798	172	2.88e-39	--	0.392	4.774e-02	N/A	N/A	N/A
☑	7	ENSG00000151718.11	WWC2	WW and C2 domain containing 2	Chr4: 184.020446	2.024	0.797	172	3.94e-39	--	-0.037	8.562e-01	N/A	N/A	N/A
☑	8	ENSG00000005810	MYCBP2	MYC binding protein 2, 2C E3 ubiquitin protein ligase	Chr13: 77.044655	2.683	0.794	172	1.48e-38	--	--	--	N/A	N/A	N/A
☑	9	ENSG00000070367.11	EXOC5	exocyst complex component 5	Chr14: 57.676285	2.435	0.793	172	2.32e-38	--	0.351	7.891e-02	N/A	N/A	N/A
☑	10	ENSG00000004700.11	RECQL	RecQ protein-like (DNA helicase Q1-like)	Chr12: 21.623903	3.303	0.774	172	1.46e-35	--	-0.239	2.395e-01	N/A	N/A	N/A
☑	11	ENSG00000267009	RP11-120M18.2	processed transcript RP11-120M18.2	Chr17: 68.413623	3.414	0.768	172	9.71e-35	--	--	--	N/A	N/A	N/A
☑	12	ENSG00000060237	WNK1	WNK lysine deficient protein kinase 1	Chr12: 0.752593	3.868	0.766	172	2.13e-34	--	0.225	2.692e-01	N/A	N/A	N/A

Step 2l: Save top 1,000 correlated gene table as CSV file

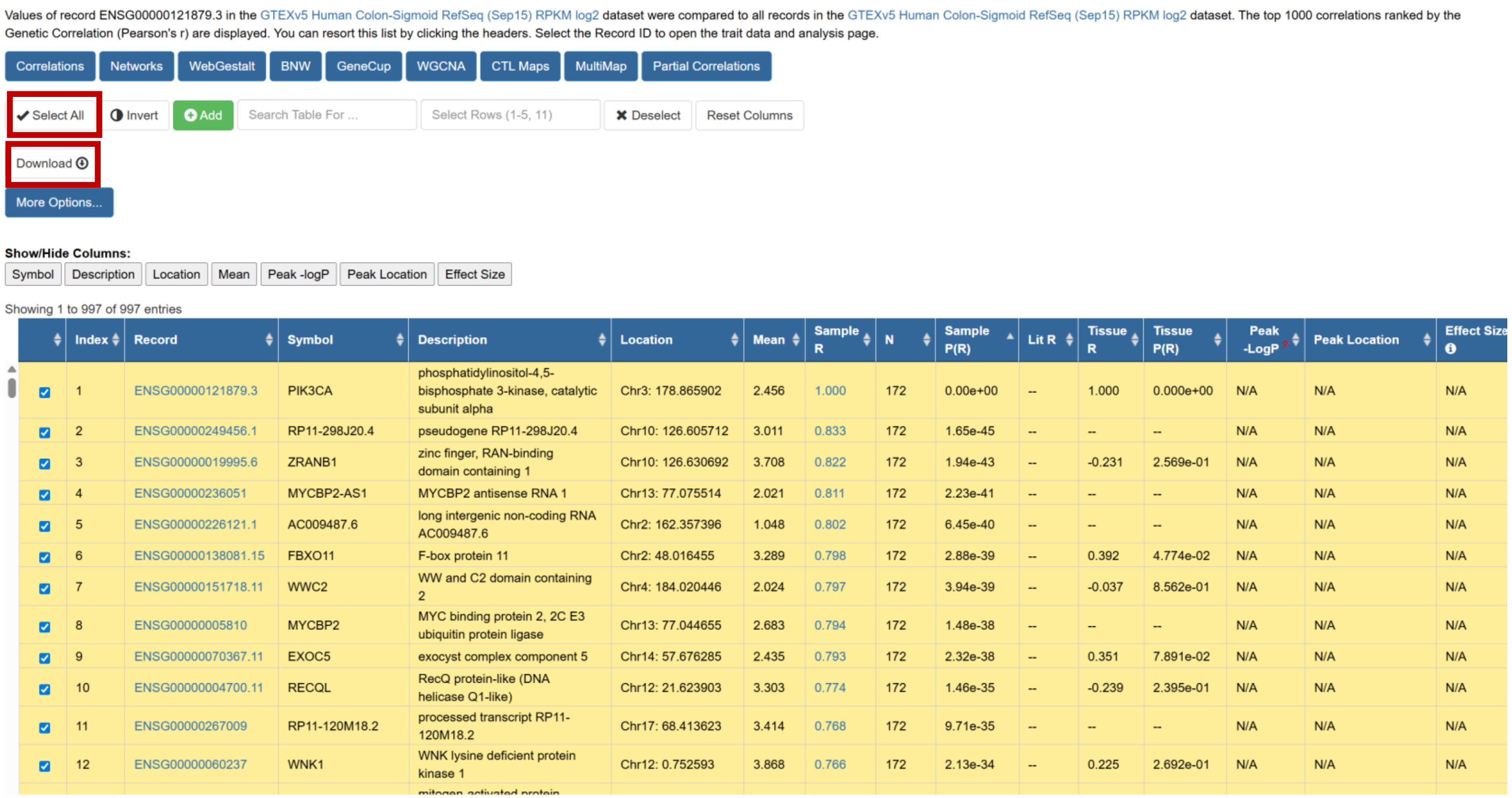

Values of record ENSG00000121879.3 in the GTEXv5 Human Colon-Sigmoid RefSeq (Sep15) RPKM log2 dataset were compared to all records in the GTEXv5 Human Colon-Sigmoid RefSeq (Sep15) RPKM log2 dataset. The top 1000 correlations ranked by the Genetic Correlation (Pearson's r) are displayed. You can resort this list by clicking the headers. Select the Record ID to open the trait data and analysis page.

Showing 1 to 997 of 997 entries

	Index	Record	Symbol	Description	Location	Mean	Sample R	N	Sample P(R)	Lit R	Tissue R	Tissue P(R)	Peak -LogP	Peak Location	Effect Size
	1	ENSG00000121879.3	PIK3CA	phosphatidylinositol-4,5-bisphosphate 3-kinase, catalytic subunit alpha	Chr3: 178.865902	2.456	1.000	172	0.00e+00	--	1.000	0.000e+00	N/A	N/A	N/A
	2	ENSG00000249456.1	RP11-298J20.4	pseudogene RP11-298J20.4	Chr10: 126.605712	3.011	0.833	172	1.65e-45	--	--	--	N/A	N/A	N/A
	3	ENSG00000019995.6	ZRANB1	zinc finger, RAN-binding domain containing 1	Chr10: 126.630692	3.708	0.822	172	1.94e-43	--	-0.231	2.569e-01	N/A	N/A	N/A
	4	ENSG00000236051	MYCBP2-AS1	MYCBP2 antisense RNA 1	Chr13: 77.075514	2.021	0.811	172	2.23e-41	--	--	--	N/A	N/A	N/A
	5	ENSG00000226121.1	AC009487.6	long intergenic non-coding RNA AC009487.6	Chr2: 162.357396	1.048	0.802	172	6.45e-40	--	--	--	N/A	N/A	N/A
	6	ENSG00000138081.15	FBXO11	F-box protein 11	Chr2: 48.016455	3.289	0.798	172	2.88e-39	--	0.392	4.774e-02	N/A	N/A	N/A
	7	ENSG00000151718.11	WWC2	WW and C2 domain containing 2	Chr4: 184.020446	2.024	0.797	172	3.94e-39	--	-0.037	8.562e-01	N/A	N/A	N/A
	8	ENSG00000005810	MYCBP2	MYC binding protein 2, 2C E3 ubiquitin protein ligase	Chr13: 77.044655	2.683	0.794	172	1.48e-38	--	--	--	N/A	N/A	N/A
	9	ENSG00000070367.11	EXOC5	exocyst complex component 5	Chr14: 57.676285	2.435	0.793	172	2.32e-38	--	0.351	7.891e-02	N/A	N/A	N/A
	10	ENSG00000004700.11	RECQL	RecQ protein-like (DNA helicase Q1-like)	Chr12: 21.623903	3.303	0.774	172	1.46e-35	--	-0.239	2.395e-01	N/A	N/A	N/A
	11	ENSG00000267009	RP11-120M18.2	processed transcript RP11-120M18.2	Chr17: 68.413623	3.414	0.768	172	9.71e-35	--	--	--	N/A	N/A	N/A
	12	ENSG00000060237	WNK1	WNK lysine deficient protein kinase 1	Chr12: 0.752593	3.868	0.766	172	2.13e-34	--	0.225	2.692e-01	N/A	N/A	N/A

Step 3b: Prepare gene list for functional annotation pathway analysis

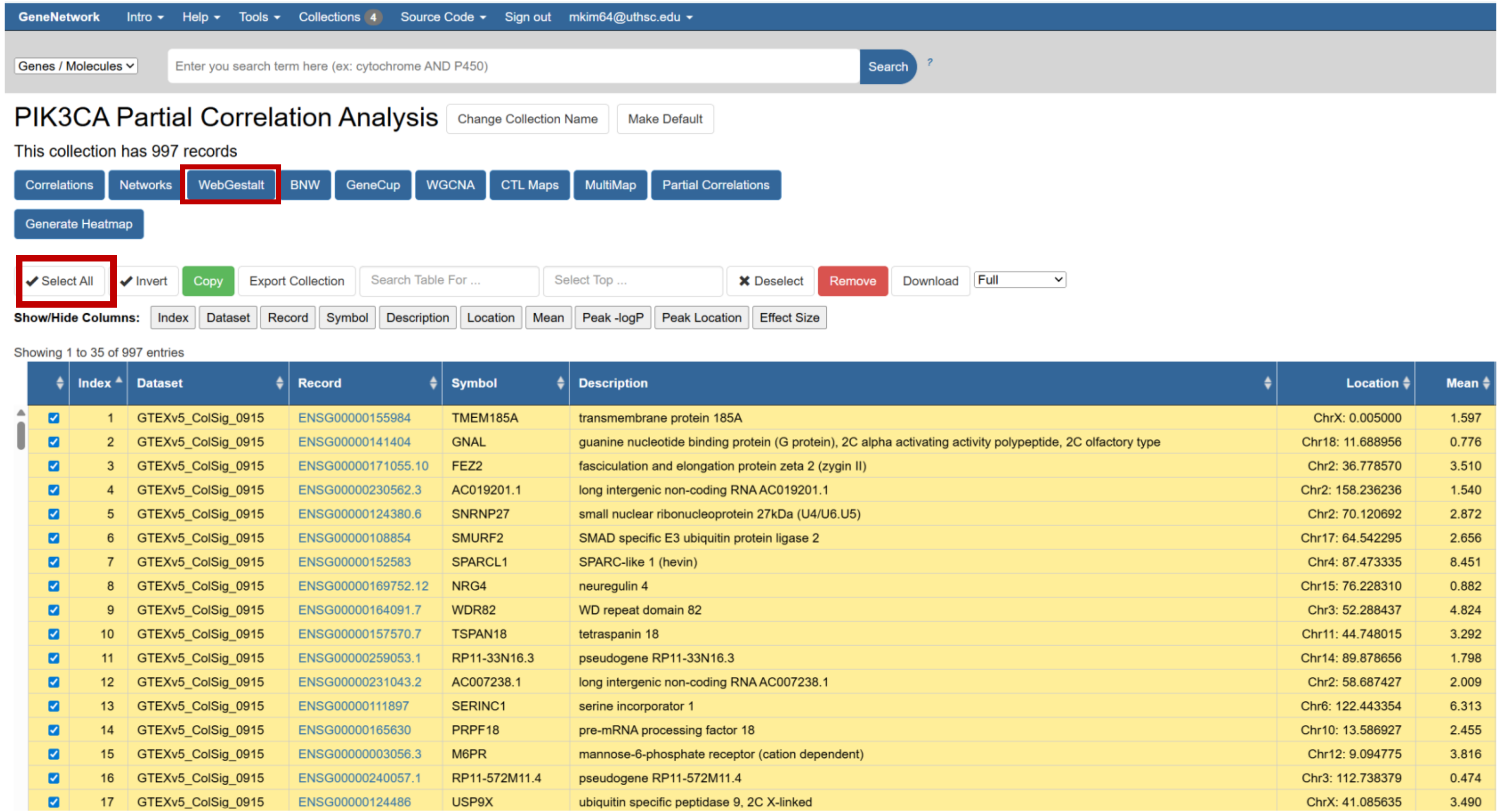

	Index	Dataset	Record	Symbol	Description	Location	Mean
☑	1	GTEXv5_ColSig_0915	ENSG00000155984	TMEM185A	transmembrane protein 185A	ChrX: 0.005000	1.597
☑	2	GTEXv5_ColSig_0915	ENSG00000141404	GNAL	guanine nucleotide binding protein (G protein), 2C alpha activating activity polypeptide, 2C olfactory type	Chr18: 11.688956	0.776
☑	3	GTEXv5_ColSig_0915	ENSG00000171055.10	FEZ2	fasciculation and elongation protein zeta 2 (zygin II)	Chr2: 36.778570	3.510
☑	4	GTEXv5_ColSig_0915	ENSG00000230562.3	AC019201.1	long intergenic non-coding RNA AC019201.1	Chr2: 158.236236	1.540
☑	5	GTEXv5_ColSig_0915	ENSG00000124380.6	SNRNP27	small nuclear ribonucleoprotein 27kDa (U4/U6.U5)	Chr2: 70.120692	2.872
☑	6	GTEXv5_ColSig_0915	ENSG00000108854	SMURF2	SMAD specific E3 ubiquitin protein ligase 2	Chr17: 64.542295	2.656
☑	7	GTEXv5_ColSig_0915	ENSG00000152583	SPARCL1	SPARC-like 1 (hevin)	Chr4: 87.473335	8.451
☑	8	GTEXv5_ColSig_0915	ENSG00000169752.12	NRG4	neuregulin 4	Chr15: 76.228310	0.882
☑	9	GTEXv5_ColSig_0915	ENSG00000164091.7	WDR82	WD repeat domain 82	Chr3: 52.288437	4.824
☑	10	GTEXv5_ColSig_0915	ENSG00000157570.7	TSPAN18	tetraspanin 18	Chr11: 44.748015	3.292
☑	11	GTEXv5_ColSig_0915	ENSG00000259053.1	RP11-33N16.3	pseudogene RP11-33N16.3	Chr14: 89.878656	1.798
☑	12	GTEXv5_ColSig_0915	ENSG00000231043.2	AC007238.1	long intergenic non-coding RNA AC007238.1	Chr2: 58.687427	2.009
☑	13	GTEXv5_ColSig_0915	ENSG00000111897	SERINC1	serine incorporator 1	Chr6: 122.443354	6.313
☑	14	GTEXv5_ColSig_0915	ENSG00000165630	PRPF18	pre-mRNA processing factor 18	Chr10: 13.586927	2.455
☑	15	GTEXv5_ColSig_0915	ENSG00000003056.3	M6PR	mannose-6-phosphate receptor (cation dependent)	Chr12: 9.094775	3.816
☑	16	GTEXv5_ColSig_0915	ENSG00000240057.1	RP11-572M11.4	pseudogene RP11-572M11.4	Chr3: 112.738379	0.474
☑	17	GTEXv5_ColSig_0915	ENSG00000124486	USP9X	ubiquitin specific peptidase 9, 2C X-linked	ChrX: 41.085635	3.490

Step 3c-g: Input parameters for Functional Annotation Pathway Analysis

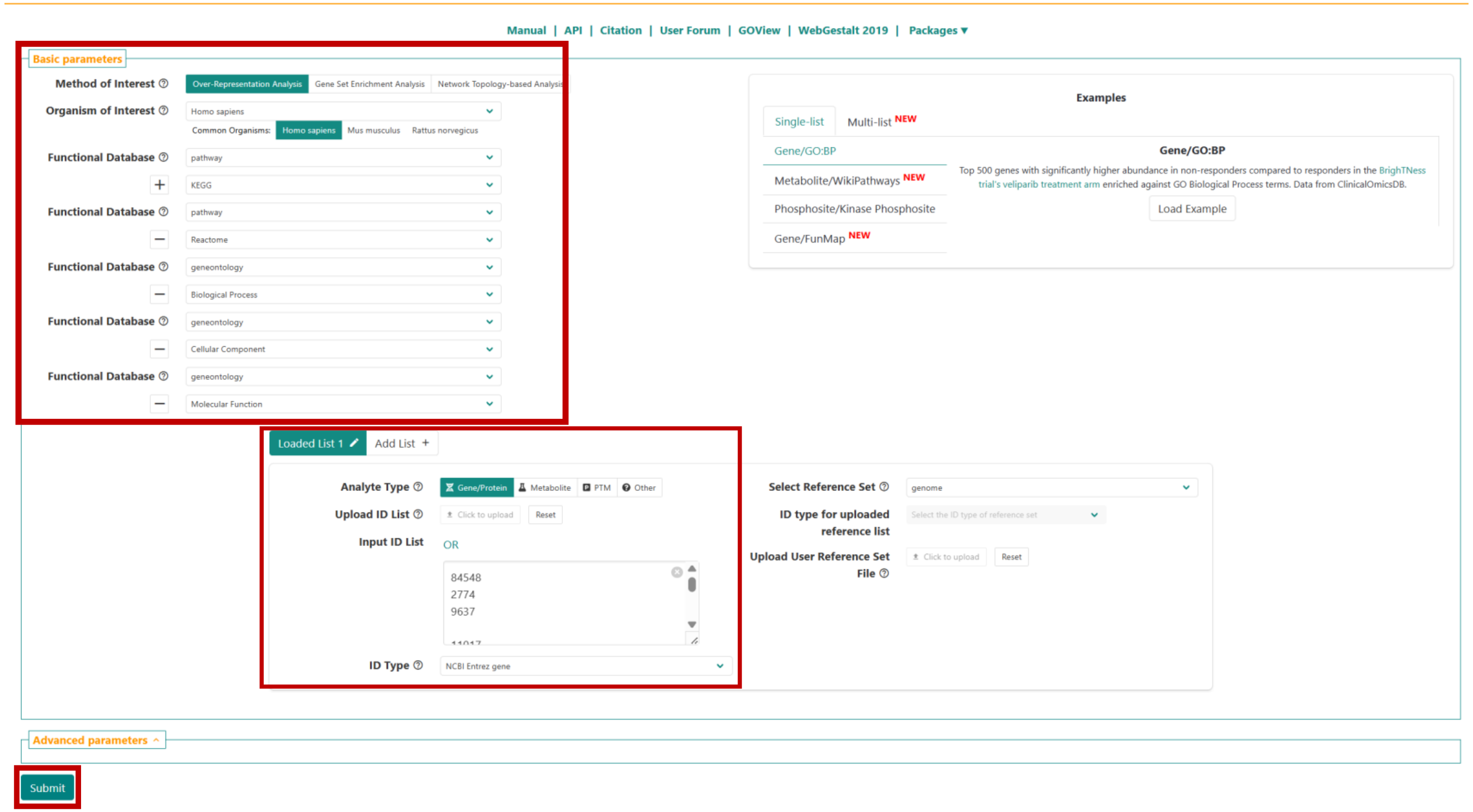

Step 4a: Access PPI network platform

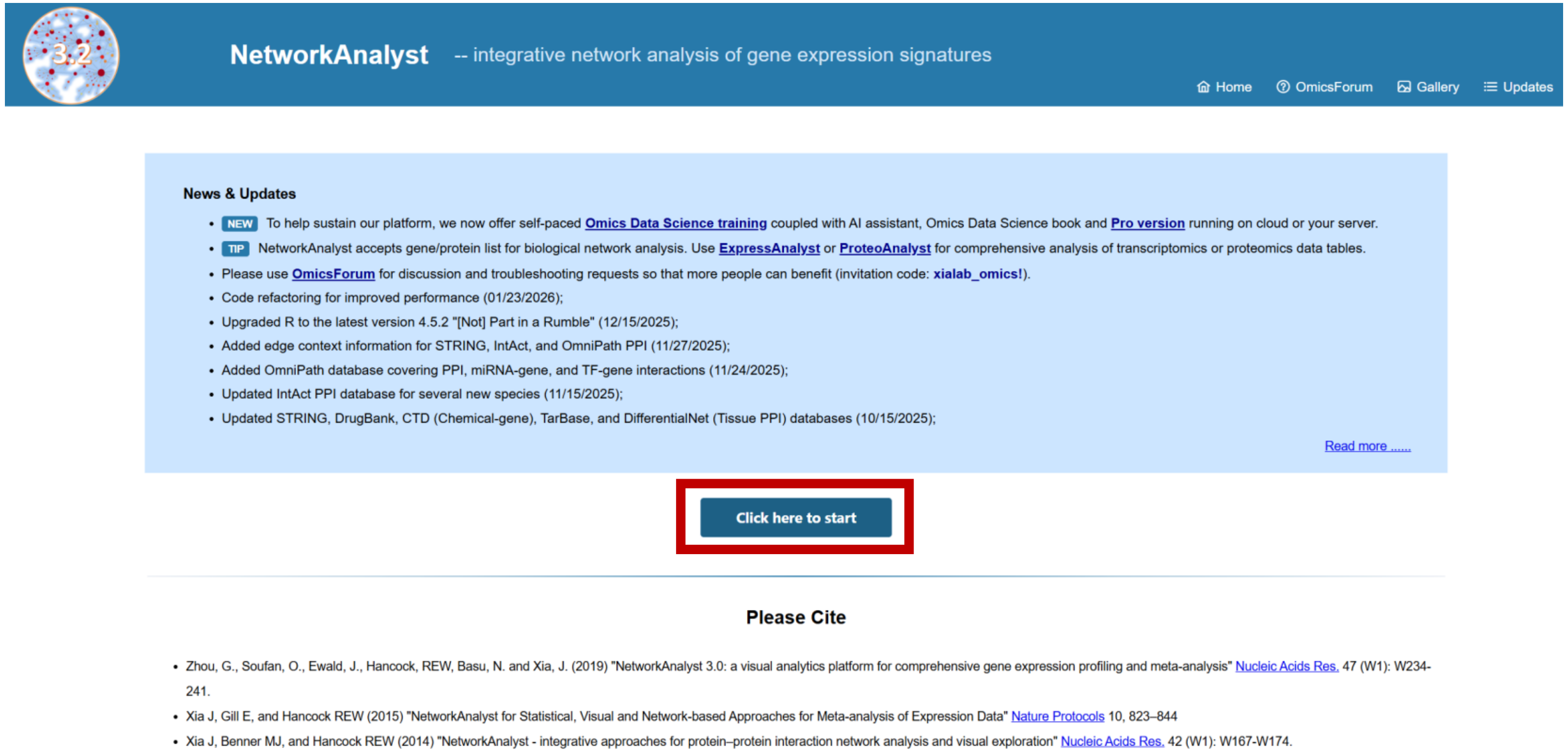

Step 3b-e: Input Parameters for PPI network generation

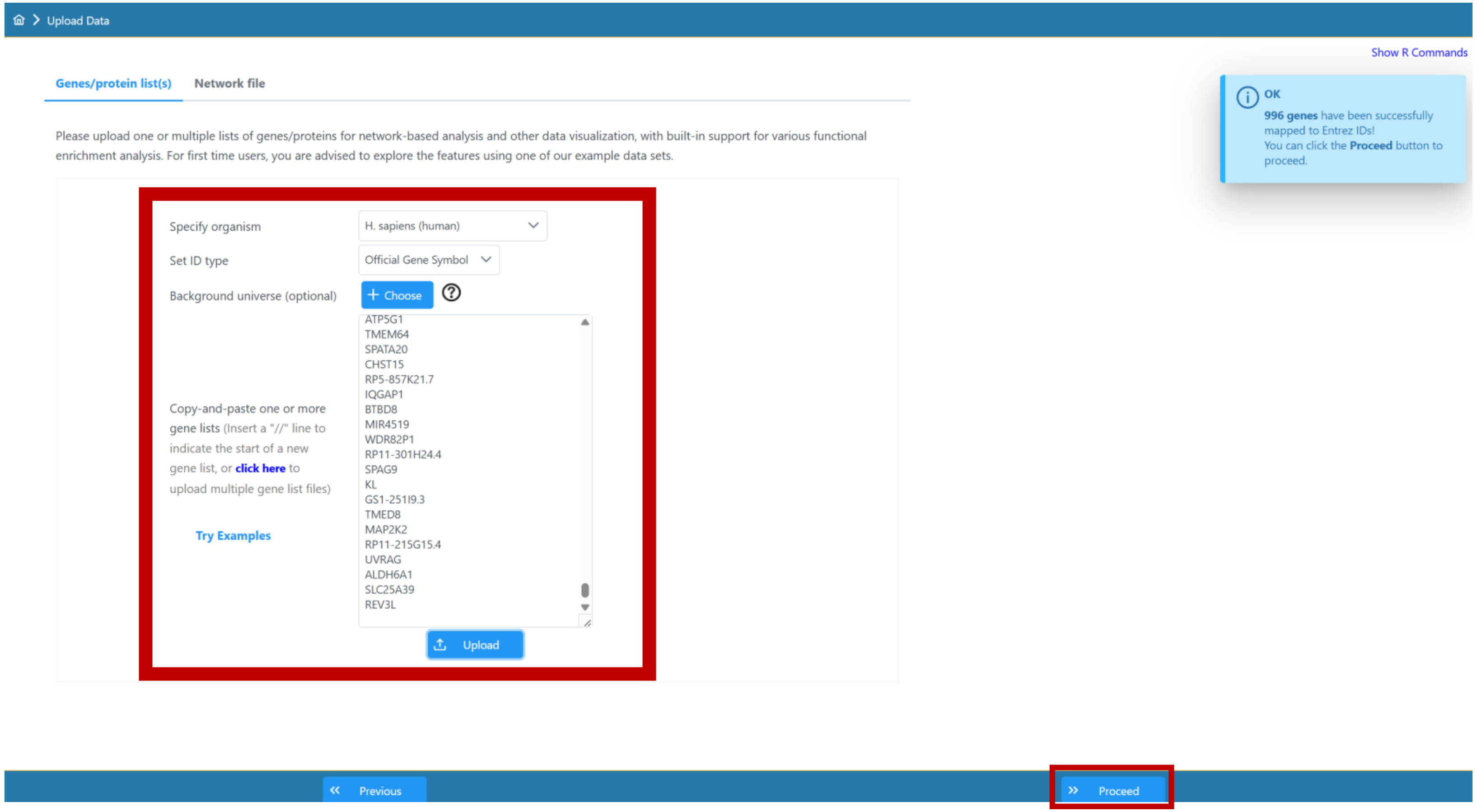

Step 4g-j: Confirm database selection for PPI network generation

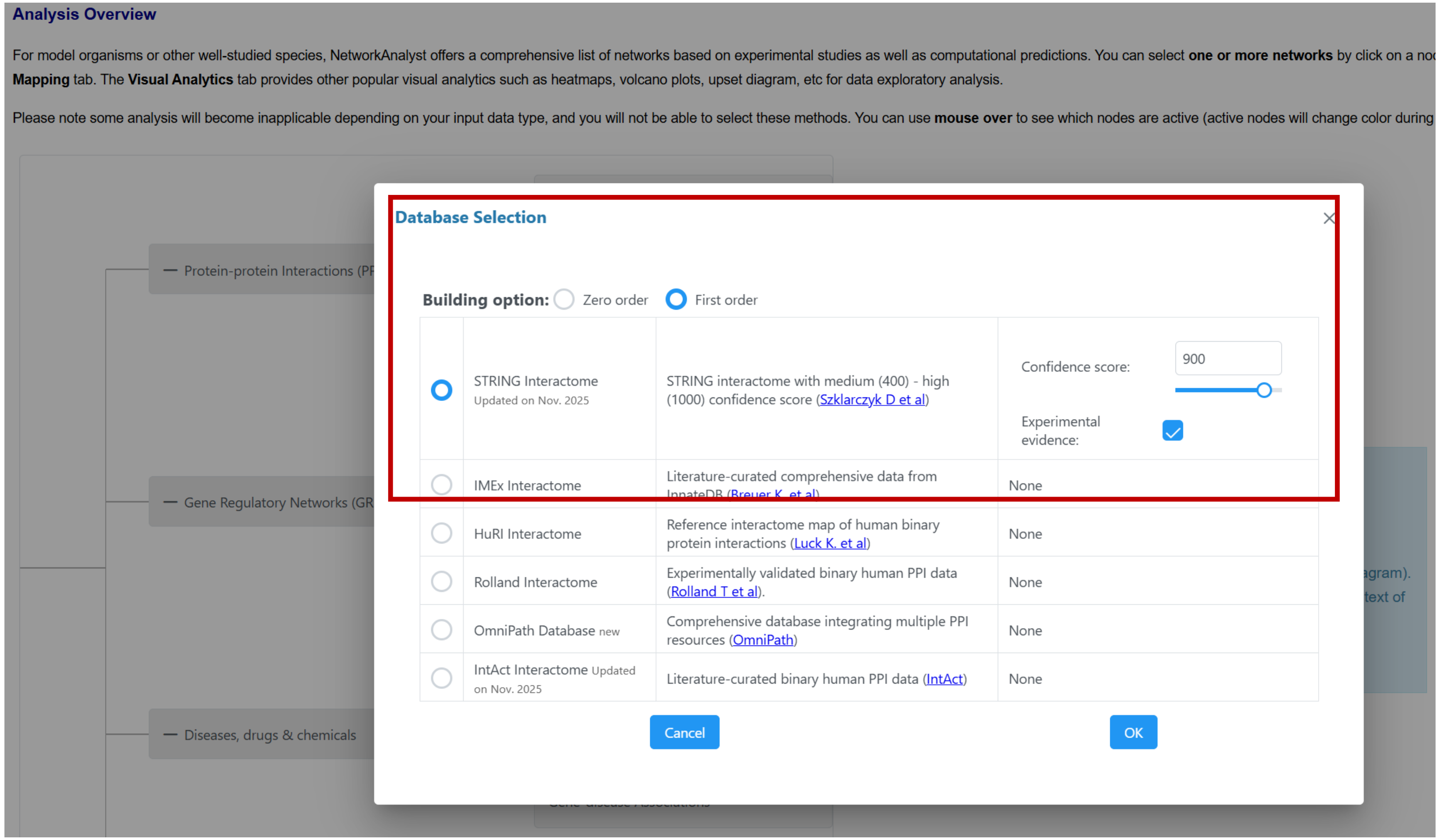

Step 4k: Selecting network setting for PPI network

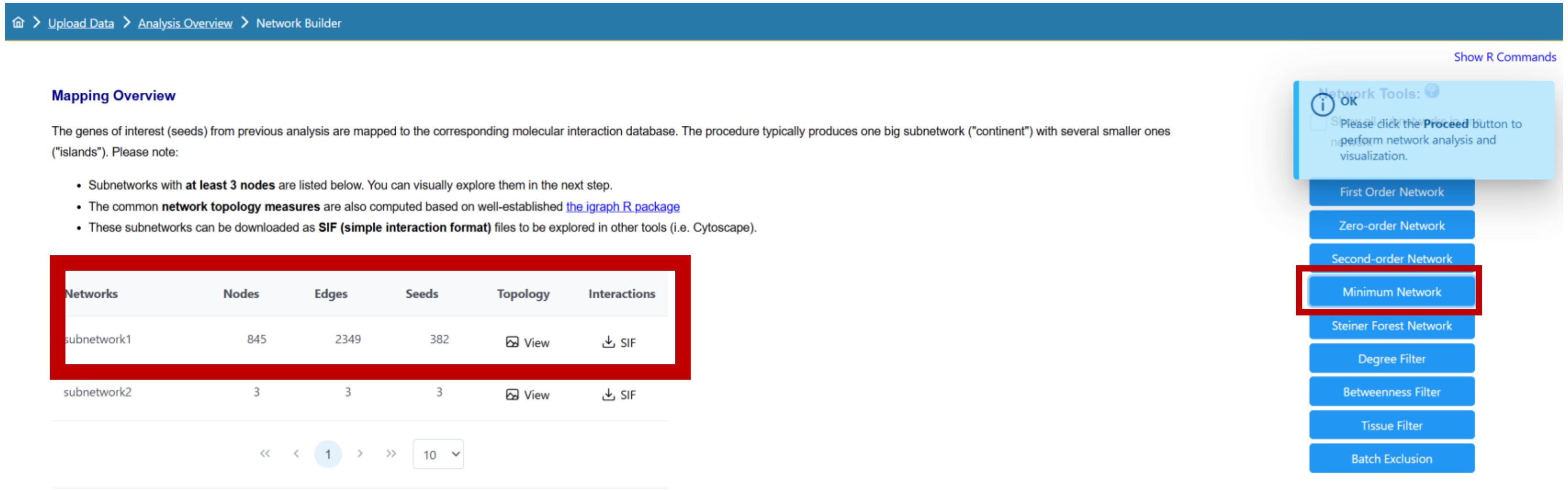

Step 4l: Visualize PPI network and download animation

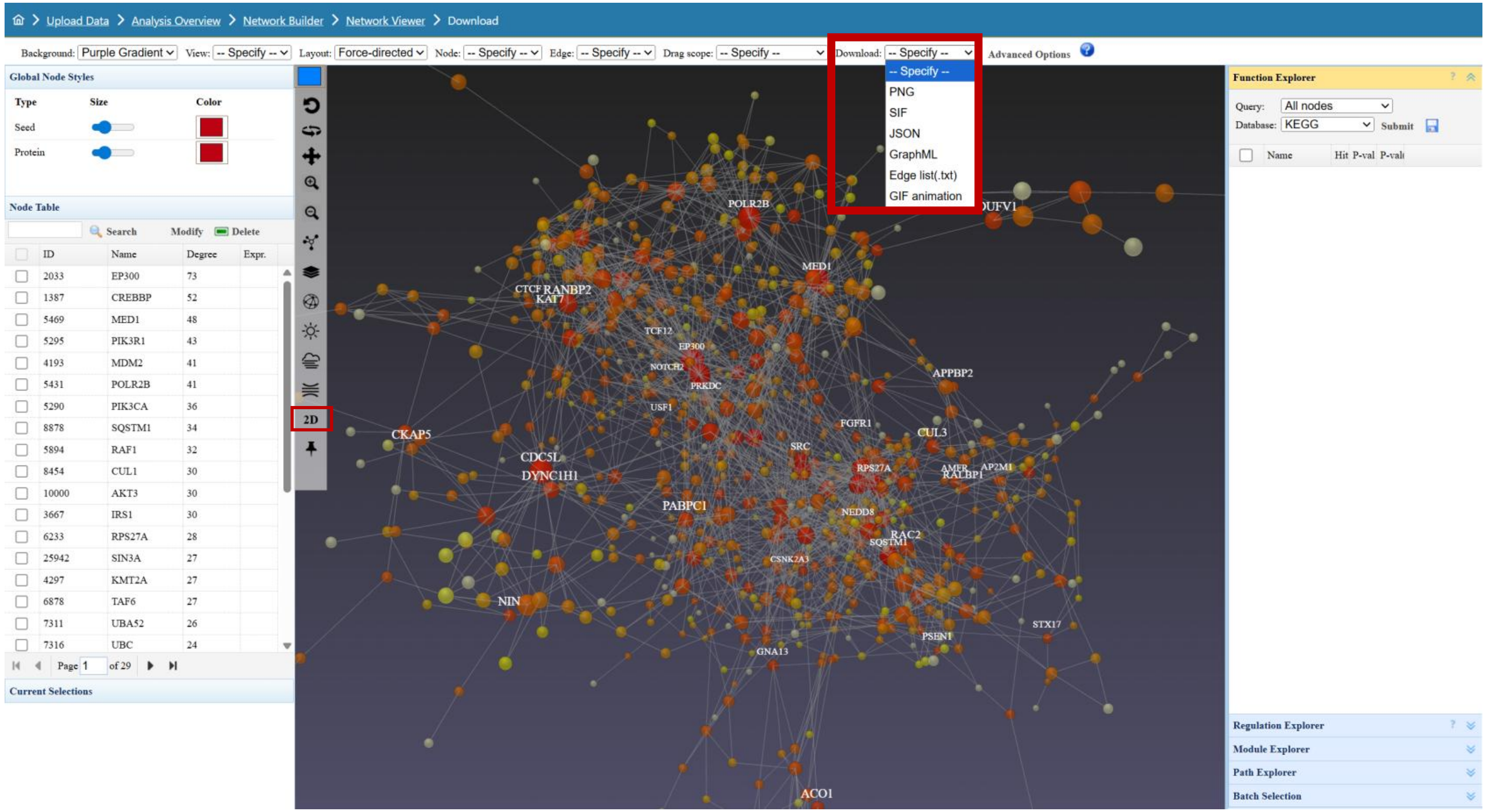

Step 4m-p: Functional annotation pathway analysis of PPI network

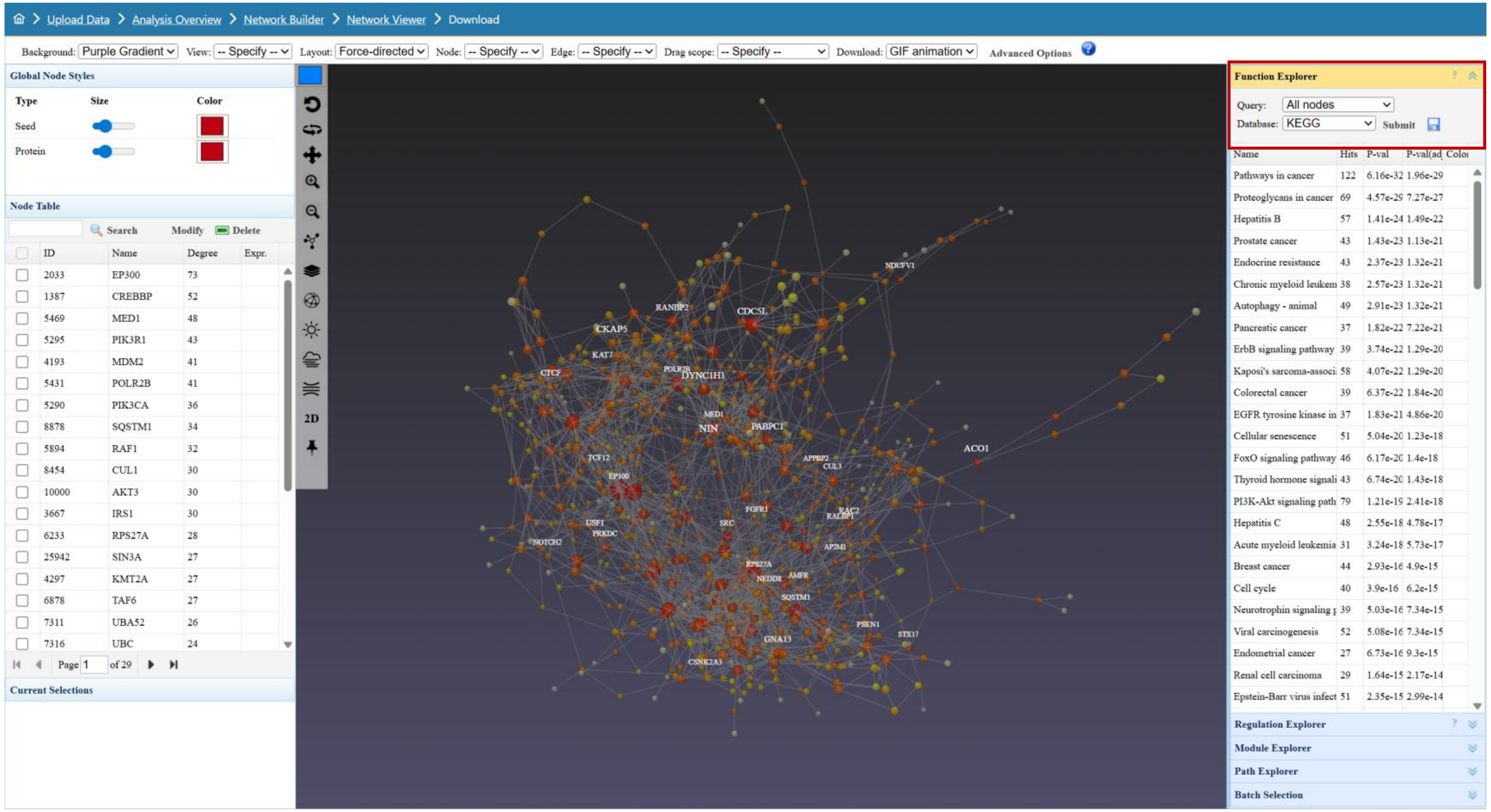

Step 5a-c: Input parameters for protein-drug interaction analysis

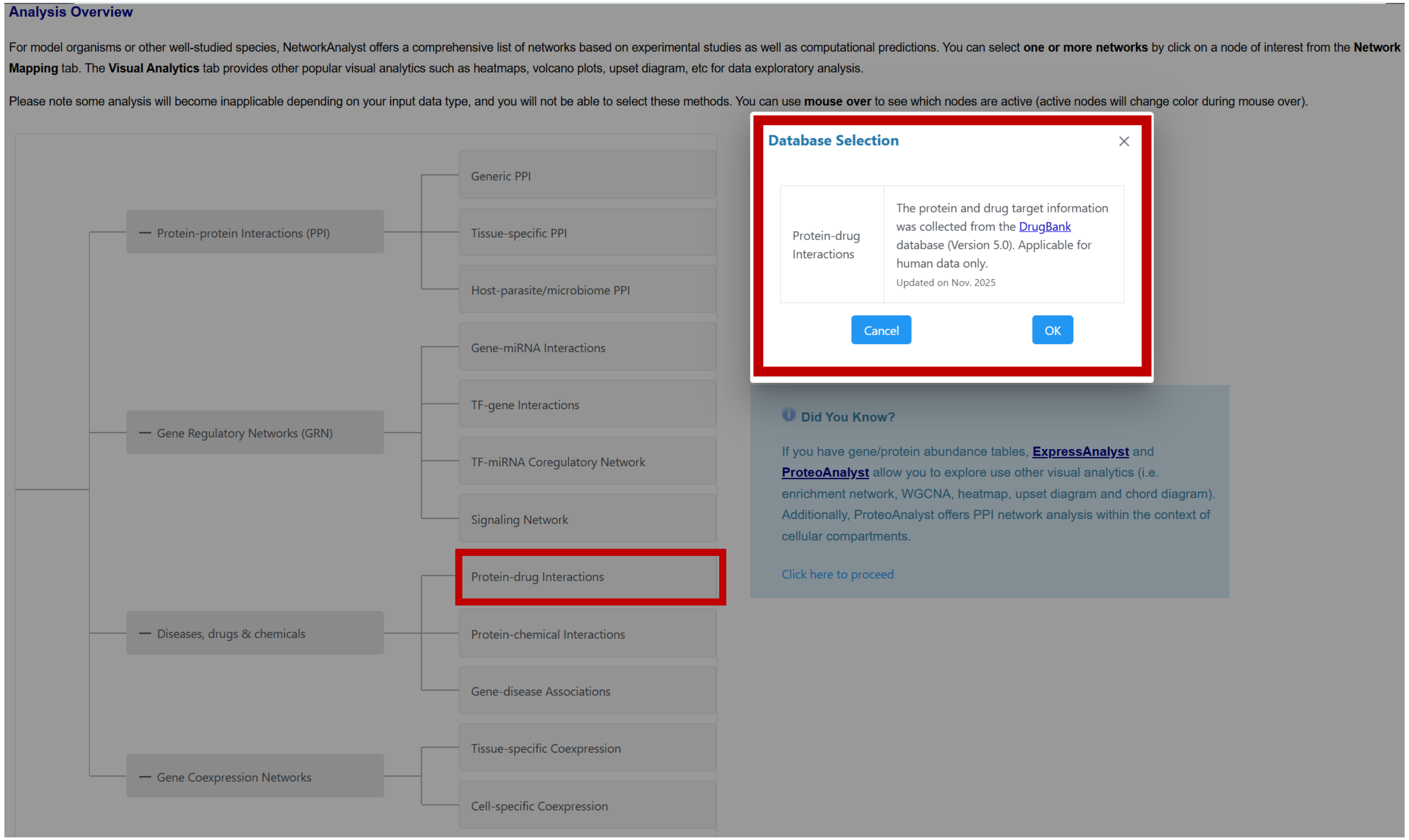

Step 5d: Select network tool for protein-drug interaction

Mapping Overview

The genes of interest (seeds) from previous analysis are mapped to the corresponding molecular interaction database. The procedure typically produces one big subnetwork ("continent") with several smaller ones ("islands"). Please note:

- Subnetworks with **at least 3 nodes** are listed below. You can visually explore them in the next step.
- The common **network topology measures** are also computed based on well-established the igraph R package
- These subnetworks can be downloaded as **SIF (simple interaction format)** files to be explored in other tools (i.e. Cytoscape).

Networks	Nodes	Edges	Seeds	Topology	Interactions
subnetwork1	117	119	26	View	SIF
subnetwork2	30	29	1	View	SIF
subnetwork3	13	12	7	View	SIF
subnetwork4	12	11	1	View	SIF
subnetwork5	12	11	1	View	SIF
subnetwork6	11	10	1	View	SIF
subnetwork7	10	11	3	View	SIF
subnetwork8	10	9	3	View	SIF
subnetwork9	10	9	1	View	SIF
subnetwork10	8	7	1	View	SIF

Network Tools:

Show all subnetworks in one network

- First Order Network
- Zero-order Network
- Second-order Network
- Minimum Network
- Steiner Forest Network
- Degree Filter
- Betweenness Filter
- Tissue Filter
- Batch Exclusion
